# Narrative Review of Nanomaterial Interactions in Plants with a Focus on Multi-Omics and Epigenetic Remodeling

**DOI:** 10.3390/plants15182802

**Published:** 2026-09-12

**Authors:** Akhil Sharma, Vikas Sharma, Shivika Sharma, Sonu Sharma, Monu Sharma, Abhishek Dadhich, Iyyakkannu Sivanesan

**Affiliations:** 1 School of Bioengineering and Biosciences, Lovely Professional University, Jalandhar 144411, India; sharmaakhil177@gmail.com (A.S.); biotech_vikas@rediffmail.com (V.S.); shivikasharma25@gmail.com (S.S.); 2 Baba Nahar Biotech and Research, Bilaspur 174001, India; sharmasonu70462@gmail.com (S.S.); monusharma311998@gmail.com (M.S.); 3 Department of Biotechnology, Graphic Era (Deemed to be University), Dehradun 248002, India; sabhi5061@gmail.com; 4 Department of Environmental Health Science, Human and Eco Care Center, Konkuk University, Hwayang-dong, Gwangjin-gu, Seoul 05029, Republic of Korea

**Keywords:** nanomaterial uptake pathways, multi-omics techniques, epigenetic changes, phytohormonal signaling pathways, metabolomic remodeling, systems-level plant–nanomaterial interactions

## Abstract

Environmental nanomaterials (ENMs) are increasingly entering agroecosystems through industrial discharges, agricultural chemicals, nanotechnology, and atmospheric deposition. Consequently, a comprehensive understanding of their interactions with plants across growth stages is essential. This narrative review synthesizes current insights into nanomaterial uptake pathways, translocation dynamics, and intracellular trafficking from seed germination to reproductive maturity. It highlights the use of integrative multi-omics techniques, namely transcriptomics, proteomics, metabolomics, and epigenomics, to elucidate molecular reprogramming in response to ENMs exposure. The data indicates that nanomaterials can significantly affect seed vigor, root architecture, photosynthetic efficiency, and other yield-related traits through coordinated regulation of stress-responsive genes, antioxidant defense mechanisms, and phytohormonal signaling pathways. Furthermore, the review underscores the role of epigenetic modifications, including DNA methylation and histone remodeling, as critical regulatory layers that govern both transient and heritable plant responses to ENMs. Metabolomic remodeling, particularly the biosynthesis of secondary metabolites and redox-related pathways, represents the primary adaptive response linking molecular disturbances to phenotypic outcomes. This manuscript proposes a systems-level framework for evaluating nano–plant interactions, bridging nanoscale physicochemical properties with physiological and yield-level outcomes. Collectively, this integrative perspective aims to enhance mechanistic clarity, support the development of predictive and sustainable nanotechnology applications in agriculture, and identify critical gaps in long-term ecological and transgenerational assessments.

## 1. Introduction

### 1.1. Emergence of Nanomaterials in Contemporary Agroecosystems

The rapid development of nanotechnology over the last two decades has substantially increased the production and application of engineered nanomaterials, leading to their widespread occurrence in agricultural and environmental systems [1]. ENMs possess unique physicochemical characteristics including elevated surface area, increased reactivity, and precisely controllable surface activity. These applications have also increased the release of engineered nanomaterials into agricultural environments. Engineered nanomaterials enter agricultural systems primarily through agricultural applications, industrial discharges, and wastewater irrigation [2]. In addition, naturally occurring and engineered nanoparticles coexist in agricultural environments, creating complex exposure conditions for plants. Therefore, contemporary agroecosystems are gradually becoming chronically exposed to multi-source nanomaterials, forming a dynamic and heterogeneous nano-environment that directly interfaces with plant life [3]. This manuscript serves as a narrative review, aiming to clarify the diverse interactions between nanomaterials and plants by focusing on the advancements in multi-omics techniques and epigenetic remodeling in response to environmental nanomaterials (ENMs). In agricultural systems, nanomaterials are introduced either intentionally or unintentionally. The use of engineered nanoparticles in fertilizers, pesticides, growth promoters, and soil conditioners is increasing to improve nutrient use efficiency and crop productivity. Unintentional inputs mainly arise from urban runoff, sewage sludge application, and industrial effluents. The result of these inputs is the accumulation of a variety of nanomaterial species in cultivated soils and irrigation waters, making plants the initial biological receptors of ENMs [4]. The perception, processing, and response of such materials by plants have thus become a key point of interest at the interface of nanotechnology, environmental science, and plant biology [5]. The environmental behavior and biological responses of engineered nanomaterials vary considerably among different nanoparticle classes, emphasizing the need to evaluate their interactions with plants within a material-specific context. 

### 1.2. Exposure Pathways and Interfaces Between Plants and Nanomaterials

Nanomaterials are continually entering plants via various interrelated routes, acting at both the subterranean and atmospheric levels. Root systems are the main entry point for soil- and water-borne nanoparticles, where intricate interactions occur within the rhizosphere microenvironment [6]. Root exudates and soil constituents modify engineered nanomaterials before plant uptake, thereby influencing their mobility and availability. Nanomaterials can enter root tissues through apoplastic spaces, cell wall pores, or active endocytic mechanisms, and ultimately via the symplastic continuum and vascular transport systems [7]. In addition to root-mediated uptake, foliar exposure is another significant pathway for aerially deposited nanoparticles. Atmospheric particulates, industrial emissions, and nano-enabled agrochemical sprays may accumulate on leaf surfaces, providing an additional pathway for plant exposure [8]. Such interactions can aid nanoparticle entry into internal leaf tissues or initiate localized stress responses. Nanomaterials, after internalization, can be translocated via the xylem and phloem networks, and thus distributed to more distant organs, such as reproductive tissues and storage organs [9]. The presented systemic mobility highlights the ability of nanomaterials to affect plant physiology across both spatial and temporal scales.

Environmental conditions strongly influence the behavior, mobility, and plant availability of nanomaterials before their uptake [10]. Nanoparticles also interact with climatic variability and agricultural management to form highly situation-specific exposure scenarios. Consequently, plant–nanomaterial interactions cannot be fully explained using simplified laboratory models, highlighting the need for integrative approaches that better represent environmental complexity [11]. Because the physicochemical properties of Ag, ZnO, TiO_2_, CeO_2_, carbon-based, and other engineered nanomaterials differ substantially, their uptake, transport, and biological responses cannot be generalized across all nanoparticle classes.

### 1.3. Weaknesses of Traditional Toxicological and Uptake Paradigms

Initial studies of the interactions between plants and nanomaterials have followed a reductionist approach to toxicology, concentrating on growth retardation, oxidative stress, membrane damage, and photosynthetic inhibition [12]. These investigations have yielded useful background data on dose-dependent phytotoxicity and short-term physiological perturbations. However, these approaches provide limited insight into the long-term molecular regulation underlying plant responses. The standard toxicity tests usually rely on high-dose experiments, which are short-lived and conducted under extremely controlled conditions in an unrealistic environment [13]. In addition, they primarily evaluate phenotypic endpoints without adequately elucidating the molecular mechanisms underlying these responses. Similarly, conventional uptake studies have largely focused on nanoparticle accumulation in plant tissues, offering limited insight into intracellular regulatory processes [14].

The methodological limitations have led to incoherent and even conflicting findings on the effects of nanomaterials on plants. Different classes of engineered nanomaterials, including Ag NPs, ZnO NPs, TiO_2_ NPs, and CeO_2_ NPs, have been reported to induce beneficial, neutral, or adverse responses under different experimental conditions [15]. These discrepancies demonstrate the failure of classical methods to explain how plant responses occur in dynamic, multilevel ways. Plants do not passively take up nanoparticles; they are active perceivers that incorporate the presence of nanoparticles into cellular signaling networks and restructure physiological and developmental programs in response to their appearance. Phenotypic observations alone cannot resolve these interconnected molecular events, underscoring the need for integrated multi-omics approaches that link cellular regulation to whole-plant responses [16].

### 1.4. Reasoning in Favor of a Multi-Omics and Life-Cycle Perspective

Recent developments in high-throughput molecular biology have revolutionized plant research by enabling profiling of changes in the expression of thousands of genes and proteins, epigenetic states, and metabolite levels [17]. Transcriptomics, proteomics, epigenomics, and metabolomics together can provide unprecedented insight into the regulatory framework that governs plant function. All these strategies can be used in an integrative approach to reconstruct the complex sets of responses to environmental cues and their relationship to phenotypic consequences [18].

Multi-omics strategies in the context of nanomaterial exposure provide an influential tool for elucidating the consequences of nanoparticle exposure on cellular homeostasis, signal transduction, and developmental programming [19]. Transcriptomics identifies changes in gene expression associated with plant responses to nanomaterial exposure. Proteomics characterizes changes in protein abundance and function associated with these responses. Epigenomics examines heritable regulatory modifications associated with stress adaptation [20]. Metabolomics captures changes in metabolic pathways associated with growth and stress responses.

**Figure 1:**
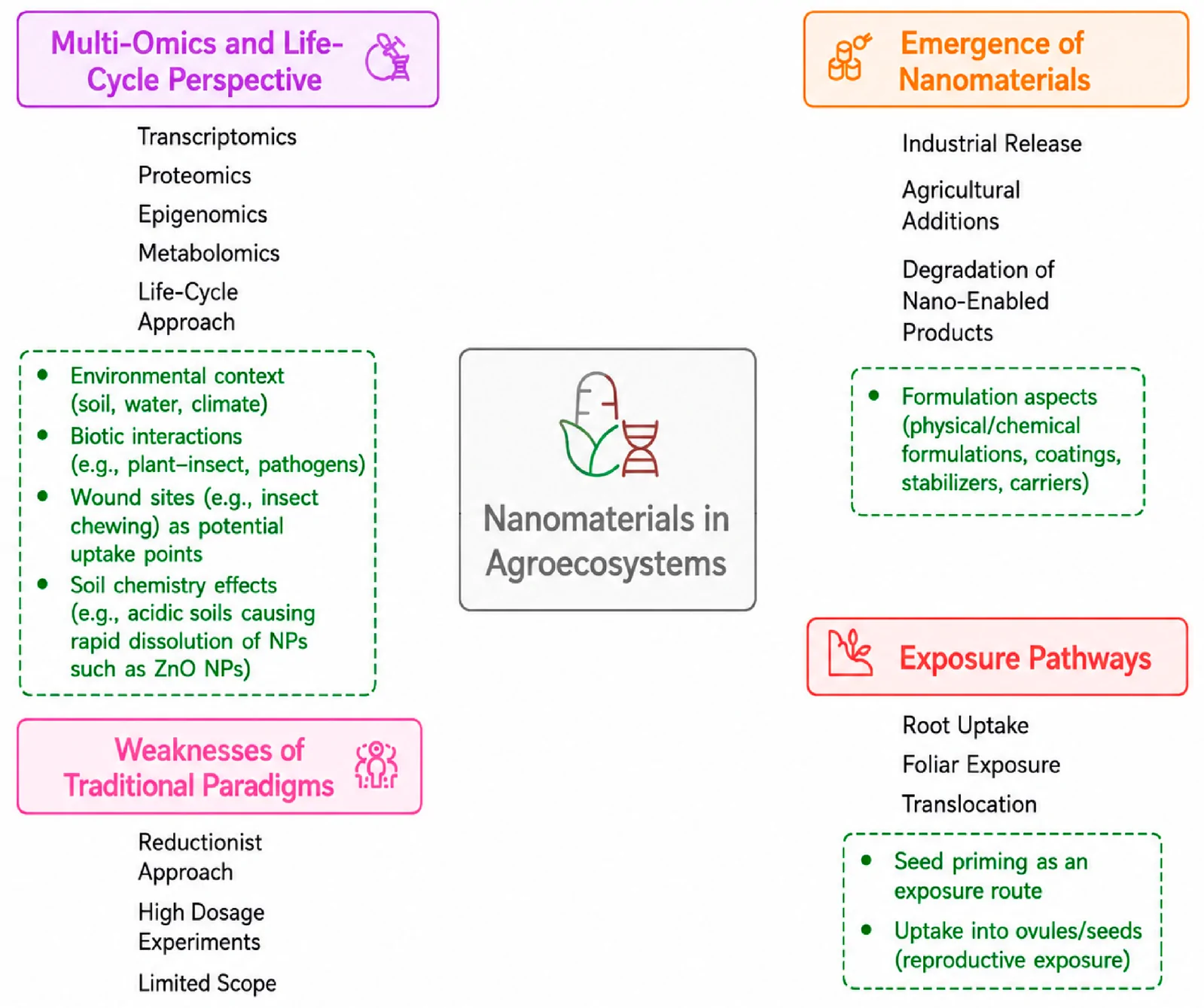
Conceptual framework illustrating the entry of nanomaterials into plants during different stages of the plant life cycle, their exposure pathways, and the resulting multi-omics responses in agroecosystems, while highlighting the limitations of traditional reductionist approaches.

It is also vital to adopt the life-cycle approach that accounts for plant responses from seed germination through reproductive maturity and yield formation. Plant responses to nanomaterials vary across developmental stages because metabolic demand, cellular differentiation, and resource allocation change throughout plant growth [20]. Exposure to nanomaterials during seed germination and seedling establishment can influence root architecture and hormonal balance, whereas exposure during the reproductive stage may affect reproductive success and grain filling. Combining omics studies across developmental stages allows determination of vulnerabilities and adaptive responses unique to each stage, offering a comprehensive view of long-term effects [21] [Figure 1].

### 1.5. Scope, Objectives, and Conceptual Positioning of the Review

The purpose of this narrative review is to synthesize existing information on plants exposed to ENMs using a multi-omics integrative approach, providing a thorough picture of the plant life cycle [22]. The review integrates evidence from uptake processes and multi-omics studies to explain how nanomaterials reshape plant regulatory networks throughout development. Particular emphasis is placed on identifying common molecular response patterns and their relationship with plant adaptation across developmental stages. Instead of viewing nanomaterials as either toxicants or agronomic tools, this review conceives of the interactions between plants and nanomaterials as properties of complex biological systems [23]. Plant responses to engineered nanomaterials arise from the integration of nanoparticle properties with developmental and environmental signals rather than from a single stress response. Consequently, the biological outcome depends on nanoparticle characteristics, plant developmental stage, and environmental conditions, resulting in either adaptive or adverse responses [24].

This narrative review presents an integrated framework linking molecular responses with plant performance and agricultural outcomes [25]. Methodological limitations, standardization challenges, and future research priorities are also discussed [26]. A systems-level and life-cycle perspective is essential for improving environmental risk assessment and the responsible application of nano-enabled agriculture [27].

Plants respond to ENMs through a sequence of interconnected events rather than isolated biological processes. Throughout this review, representative engineered nanomaterials, including Ag NPs, ZnO NPs, TiO_2_ NPs, CeO_2_ NPs, and carbon-based nanomaterials, are discussed separately where their biological behavior differs. The physicochemical characteristics of engineered nanomaterials determine their behavior during plant exposure. These initial interactions influence cellular recognition, uptake efficiency, intracellular trafficking, and tissue distribution, thereby shaping subsequent molecular responses. Consequently, molecular responses observed through multi-omics analyses should be interpreted in the context of these early plant–nanomaterial interactions. Accordingly, this review follows a mechanistic framework that links nanomaterial properties with molecular reprogramming and ultimately with plant growth, productivity, and environmental adaptation. Special attention is given to the relationship between molecular regulation and changes in plant metabolism that influence crop performance and quality.

The biological effects of engineered nanomaterials depend on exposure dose, nanoparticle properties, plant species, and environmental conditions. Depending on nanoparticle characteristics, exposure intensity, plant genotype, and environmental conditions, the same nanomaterial may either promote adaptive responses or induce oxidative damage, metabolic disruption, and growth inhibition. This narrative review therefore considers both beneficial and adverse outcomes within a unified mechanistic framework.

## 2. Nanomaterials Classification and Environmental Fate of Nanomaterials in Plant Habitats

Nanomaterials used in agriculture encompass a wide range of compositions and structures with distinct physicochemical properties that influence their interactions with plants and soil systems. In addition to metallic nanoparticles such as silver (Ag), copper (Cu), iron (Fe), and gold (Au), metal oxide nanoparticles including zinc oxide (ZnO), titanium dioxide (TiO_2_), cerium oxide (CeO_2_), iron oxide (Fe_3_O_4_), and silicon dioxide (SiO_2_) have been widely investigated for their effects on plant growth, nutrient delivery, and stress tolerance. Carbon-based nanomaterials, such as carbon nanotubes, graphene oxide, fullerenes, and carbon dots, have attracted considerable attention because of their unique structural and transport properties. Rather than their source alone, nanoparticle composition, size, surface charge, morphology, dissolution behavior, and surface chemistry determine their environmental fate and biological responses in plants. Therefore, this review discusses nanomaterials based on their material-specific properties rather than treating all nanoparticles as a single group.

Different classes of nanomaterials exhibit distinct modes of action in plant systems. For example, ZnO and Fe_3_O_4_ nanoparticles are commonly investigated as nutrient sources; TiO_2_ nanoparticles have frequently been associated with improvements in photosynthetic efficiency; SiO_2_ nanoparticles are widely studied for enhancing tolerance to abiotic stresses; and chitosan nanoparticles and polymer-based nanocarriers have emerged as promising delivery platforms for agrochemicals and biomolecules. Similarly, graphene oxide, carbon nanotubes, and carbon dots have been explored for their roles in seed germination, nutrient transport, and plant stress responses. These examples illustrate that nanoparticle effects cannot be generalized across all nanomaterials as their biological activity strongly depends on their chemical composition and physicochemical characteristics.

### 2.1. Diversity of Nanomaterials in Terrestrial and Agricultural Environments

The sources of nanomaterials in natural and anthropogenic environments are diverse, and thus the resulting physicochemical profiles are highly heterogeneous, affecting how they behave in the environment and interact with living organisms [28]. Metallic and metal oxide nanoparticles are the most commonly used, widely utilized, and environmentally prevalent engineered nanomaterials [29]. When these nanoparticles are released into agroecosystems, their interactions with soil components, plant exudates, and microbial communities are dynamic, so their surface properties are continuously changing [30].

Although ENMs originate from diverse natural and engineered sources, their biological behavior in plants is determined less by their origin than by their physicochemical characteristics [31]. Variations in particle size, morphology, surface charge, surface chemistry, aggregation behavior, and dissolution potential influence how nanomaterials interact with root surfaces, penetrate biological barriers, and move through plant tissues [32].

Plant responses are determined primarily by nanoparticle physicochemical properties, including size, morphology, surface chemistry, dissolution behavior, and surface coatings [33]. Consequently, different nanomaterials may induce distinct molecular and physiological responses despite sharing similar environmental sources, emphasizing that physicochemical identity is the primary determinant of plant–nanomaterial interactions rather than material origin alone [34].

**Figure 2:**
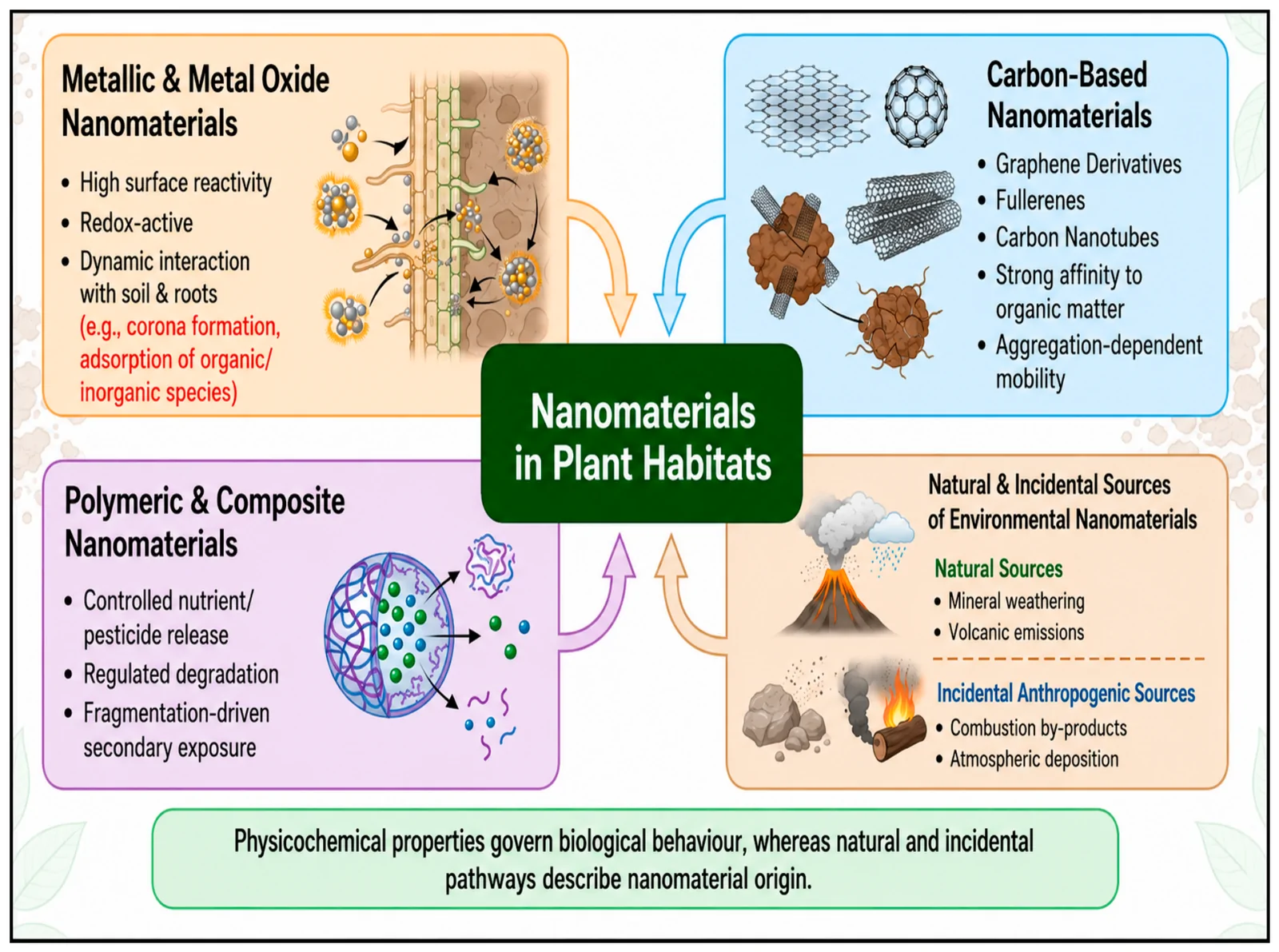
Classification of environmental nanomaterials in plant habitats based on composition (metallic and metal oxide, carbon-based, and polymeric/composite nanomaterials) and origin (natural and incidental sources), highlighting that physicochemical properties primarily govern their biological interactions with plants. The arrow depicts the pathway and movement of nanomaterials in the plant-soil system. For metallic and metal oxide nanomaterials, the arrow indicates their interaction with soil and the root surface, followed by uptake and translocation within the plant.

Consequently, plants are simultaneously exposed to multiple nanoparticle classes that differ in their environmental behavior and biological activity [35] [Figure 2]. These naturally occurring nanosized particles coexist with engineered nanomaterials and may exhibit comparable size ranges and surface properties. For example, clay mineral nanoparticles and natural silica colloids can resemble engineered SiO_2_ nanoparticles, while naturally occurring iron oxide nanoparticles share physicochemical characteristics with engineered iron oxide nanomaterials. Mixed assemblages of physicochemically similar natural and engineered nanoparticles also complicate environmental monitoring and risk assessment because plants are simultaneously exposed to overlapping nanoparticle assemblies [36]. Therefore, classifying nanomaterials only by their origin provides limited biological information. Their physicochemical properties are more important for determining plant uptake, molecular responses, and omics-based changes discussed in the following sections.

### 2.2. Agroecosystems Environmental Transformation and Aging Processes

When nanomaterials are introduced into agricultural settings, they undergo significant physicochemical changes due to biotic and abiotic processes [37]. The changes gradually alter particle size, surface charge, solubility, and reactivity, thereby redefining their interactions with plant systems. Aggregation and disaggregation determine nanoparticle mobility and availability in soil, thereby influencing their interaction with plant roots [38]. Electrostatic screening encourages the clustering of particles in soils with high ionic strength or high concentrations of divalent cations, thereby lowering their surface area and transport capacity. On the other hand, the dispersed states may be stabilized by association with organic ligands and root exudates, thereby enabling long-distance transport [39].

Dissolution is particularly important for ZnO, CuO, and Ag nanoparticles, which can release bioactive ions under acidic or oxidative conditions. Dissolution produces both nanoparticles and released metal ions, which may trigger different biological responses [40]. These coupled forms can trigger distinct cellular signaling pathways, making their mechanistic interpretation difficult. Simultaneously, adsorption of proteins, polysaccharides, humic substances, and microbial metabolites develops dynamic coronas on the surface that re-identify nanoparticles [41]. This eco-corona regulates subsequent interactions with the root membranes, transport proteins, and intracellular compartments. Interactions with soil organic matter and microorganisms further modify nanoparticle surfaces and behavior. Microbial biofilms can immobilize particles, facilitate redox reactions, or catalyze surface modification [42].

Environmental conditions continuously modify nanoparticle behavior after their release into agricultural systems [43]. These processes cause structural flaws, surface oxidation, and fragmentation, resulting in secondary nanoparticles with modified biological characteristics. The environmentally driven transformations continuously modify the biological identity of nanomaterials before they encounter plant tissues. Environmental aging alters nanoparticle surface chemistry, aggregation, and stability, thereby changing their interactions with plants [44]. Consequently, nanomaterials that have undergone environmental or biological transformation often exhibit physicochemical properties and biological responses that differ from those of pristine nanomaterials [45]. These transformations determine the physicochemical form in which nanoparticles reach plant tissues and, in turn, influence the molecular responses discussed in the following omics sections.

### 2.3. Bioavailability and Exposure Dynamics, Plant Systems

Nanomaterial bioavailability depends on nanoparticle properties, soil conditions, and plant physiology [46]. The rhizosphere is a highly dynamic interface that integrates chemical gradients, biological activity, and physical heterogeneity to regulate nanoparticle accessibility. Nanoparticle speciation in the rhizosphere is shaped by root exudates, such as organic acids, amino acids, phenolics, and polysaccharides, together with local redox processes that influence their dissolution, transformation, and oxidation state [47]. These compounds can chelate metal ions, stabilize dispersed particles, or promote aggregation depending on their concentration and molecular composition. In addition, humic fractions, including humic and fulvic acids, interact with nanoparticle surfaces, modifying their surface charge, aggregation behavior, and metal complexation. Together, root exudates and soil organic matter continuously modify the availability of nanoparticles before plant uptake [48].

Nanoparticle retention and transport are also affected by soil texture and mineralogy. Clay-rich soils generally retain nanoparticles, whereas sandy soils promote their movement [49]. Moisture regimes control pore connectivity and diffusion rates, thereby determining how nanoparticles are delivered to root surfaces. When exposed to waterlogged conditions, redox variations can trigger dissolution and reprecipitation cycles, thereby altering exposure profiles [50].

In addition to root-mediated pathways, irrigation practices play an important role in determining nanoparticle distribution within agricultural systems. Nanoparticles and nanoplastics can be introduced into cropping systems through reclaimed wastewater, surface water, nano-enabled fertilizers, and other agricultural inputs, resulting in variable exposure levels across different environmental conditions [51]. Irrigation water, reclaimed wastewater, and surface runoff can redistribute nanoparticles within the soil, thereby affecting their movement toward plant roots and subsequent uptake. A separate exposure pathway involves foliar deposition, in which nanoparticles are delivered via atmospheric fallout, aerosolized agrochemicals, or resuspended dust. Their retention and penetration are further influenced by leaf surface characteristics, including cuticle thickness, trichome density, and stomatal architecture [52]. Climatic and seasonal influences significantly impact exposure dynamics. Temperature also controls the rates of reactions, microbial activity, and plant metabolism, thereby determining nanoparticle transformation and uptake. Rainfall patterns influence leaching, runoff, and resuspension, whereas drought conditions shrink the soil pore network and increase particle concentrations [53]. Phenological stage also interacts with climatic conditions, as developmental changes modify root architecture, transpiration rates, and nutrient requirements. Together, environmental conditions create dynamic exposure patterns that influence nanoparticle uptake and subsequent molecular responses in plants [54]. The extent of nanomaterial bioavailability is not determined solely by the quantity of particles reaching plant surfaces but also by the physicochemical form in which they are presented to the plant. Environmental conditions regulate particle stability, surface characteristics, and mobility, thereby influencing cellular recognition and uptake pathways. These early interactions determine how plant cells recognize nanoparticles and form the basis of the transcriptomic, proteomic, metabolomic, and epigenetic responses discussed in the following sections. Because these exposure dynamics differ among Ag NPs, ZnO NPs, TiO_2_ NPs, CeO_2_ NPs, and other engineered nanomaterials, uptake patterns should be interpreted in relation to nanoparticle identity rather than generalized across all ENMs.

### 2.4. Association of Environmental Transformation and Molecular Uptake Behavior

One of the major challenges in understanding plant–nanomaterial interactions is linking environmental transformations of nanomaterials to their intracellular responses. The particles entering plant tissues are rarely pristine; instead, they have often undergone aggregation, surface corona formation, partial dissolution, or changes in chemical composition during environmental exposure. These transformations are particularly important for ZnO, Ag, CuO, and metal sulfide nanoparticles because they modify particle uptake, transport, and biological responses in plants [55]. These altered identities dictate membrane receptor and transporter recognition, as well as endocytic recognition, thereby affecting cellular entry pathways and subcellular localization. Multi-omics analyses of the recent past have shown that minor changes in surface chemistry and dissolution profiles can lead to unique transcriptional, epigenetic, and metabolic alterations [56]. Biomolecules associated with the eco-corona may either facilitate or reduce nanoparticle uptake by modifying interactions with the plant cell surface [52]. Therefore, environmental characterization together with multi-omics analysis provides a mechanistic link between nanoparticle transformation and plant molecular responses [57].

Combining environmental characterization with omics analysis allows environmental processes to be directly linked with intracellular molecular responses. It enables the identification of transformation-dependent biomarkers and predictive descriptors that relate ecosystem processes to molecular phenotypes [58]. Such knowledge is essential for developing robust frameworks for nano-risk monitoring, guiding sustainable nanomaterial design, and supporting precision agriculture. The biological outcome depends on nanoparticle identity, transformation state, and exposure concentration. Consequently, different nanomaterials may promote adaptation or induce phytotoxicity depending on their physicochemical characteristics and exposure conditions. Accordingly, environmental fate should be regarded as the mechanistic foundation upon which subsequent multi-omics responses are established.

## 3. Plant Developmental Windows of Nanomaterial Sensitivity

Plant responses to nanomaterials vary with developmental stage because cellular differentiation, metabolism, and regulatory networks change throughout the life cycle [59]. Accordingly, the biological effects of Ag NPs, ZnO NPs, TiO_2_ NPs, CeO_2_ NPs, and other engineered nanomaterials may differ substantially across developmental stages. These developmental windows affect not only short-term physiological outcomes but also long-term growth patterns and reproductive functioning [60]. Recognizing these stage-specific responses provides the biological context for interpreting the transcriptomic, proteomic, epigenomic, and metabolomic changes discussed in subsequent sections.

### 3.1. Sensitivity During Seed Imbibition and Germination

The germination period is a critical life stage that marks the transition from metabolic quiescence to active development, during which seeds undergo rapid rehydration, enzyme activation, and hormonal homeostasis [61]. The major elements controlling solute and nanoparticle uptake during imbibition are the seed coat and related mucilage layers. The interaction of nanomaterials with these protective structures is determined by their physicochemical properties, such as size, surface charge, and hydrophobicity [62]. Adsorption onto seed surfaces or through microfissures can alter the permeability distribution, thereby influencing seed permeability during imbibition. During germination, nanoparticle exposure can modify hormone signaling and cellular redox homeostasis that regulate seedling establishment [63]. Exposure to ZnO, SiO_2_ and carbon-based nanoparticles has been reported to modify redox balance and hormone signaling during germination, although the response depends on nanoparticle identity and exposure level. Moderate changes in hormone signaling may enhance germination, whereas excessive disturbance can delay germination and reduce seedling vigor. In addition, transcriptomic and epigenetic modifications initiated during germination may persist beyond germination, influencing subsequent growth and developmental responses [64].

### 3.2. Seedling Establishment and Remodeling of the Root System

**Figure 3:**
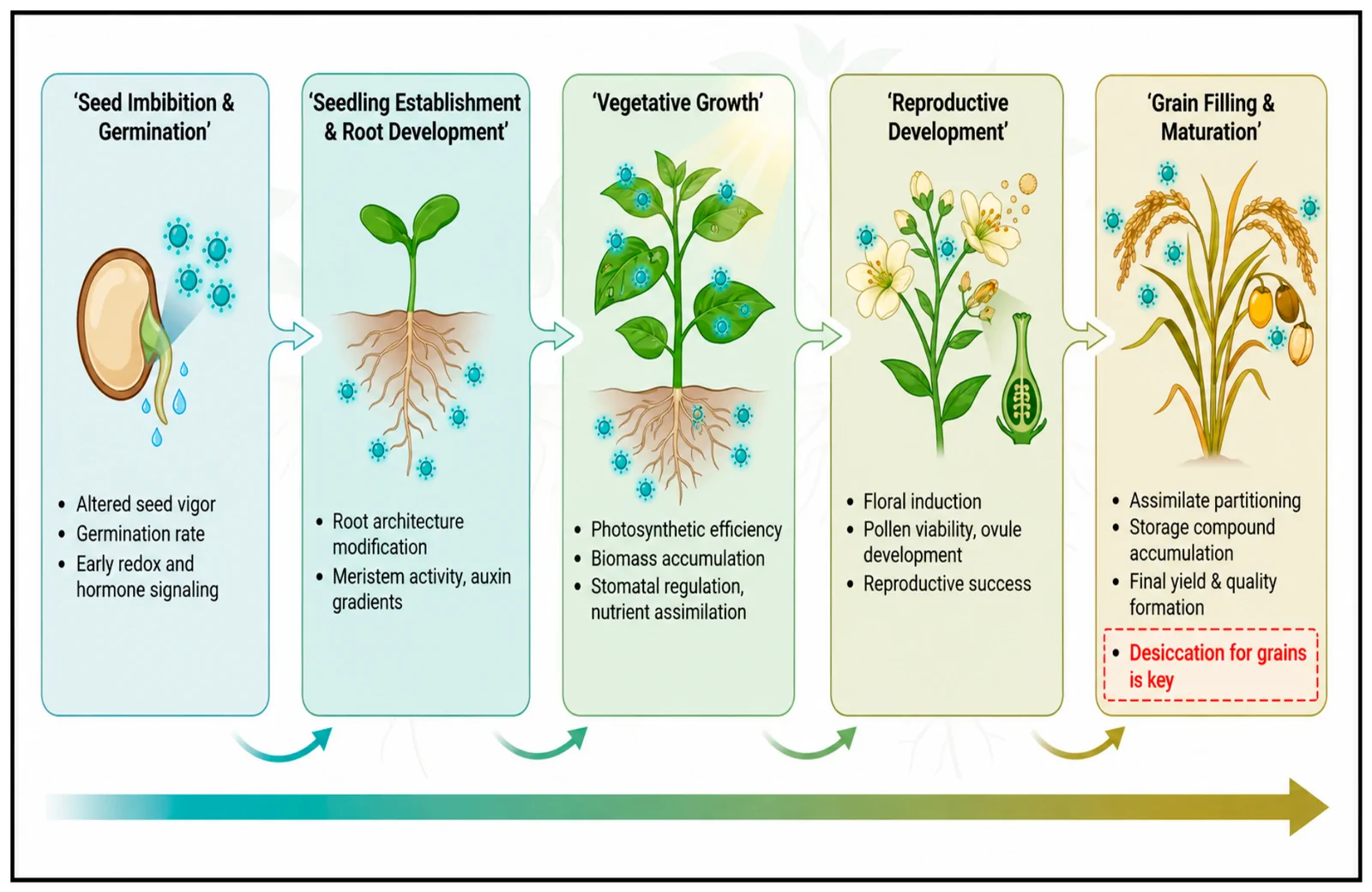
Schematic representation of plant developmental windows sensitive to nanomaterial exposure, highlighting stage-specific effects from seed imbibition to grain filling.

After germination, seedlings undergo rapid structural differentiation and optimize resource acquisition, with root systems as key actors in environmental sensing and nutrient maintenance [65] [Figure 3]. The apical meristem of the root and the elongation zone are highly susceptible to external disturbances because of their high mitotic activity and metabolic flux. Accumulation of ZnO, Ag, and CuO nanoparticles around the root tip may alter cell division, cytoskeletal organization, and membrane trafficking, thereby affecting root meristem activity [66]. Several nanoparticles, including ZnO nanoparticles and carbon nanotubes, have been reported to modify auxin transport, although the response depends on nanoparticle identity and exposure conditions [67]. These structural changes influence subsequent resource acquisition and plant establishment. At the same time, proteomic and metabolomic results suggest that early exposure commonly reprograms primary metabolism and antioxidant systems, which represent adaptive responses to remodeled cellular redox speciation [68,69].

### 3.3. Vegetative Growth and Reproductive Development

During the vegetative growth stage, plants invest substantial resources in developing leaf area, vascular differentiation, and photosynthetic capacity. Foliar application of TiO_2_, ZnO, and SiO_2_ nanoparticles has been reported to influence photosynthesis, stomatal conductance, and nutrient assimilation, although responses differ among nanomaterials and plant species [70]. The transition to reproduction is regulated by hormonal, photoperiodic, and epigenetic pathways that may be influenced by nanoparticle exposure. Nanoparticle-induced molecular perturbations during this transition may influence floral induction and reproductive development. Translocation of nanoparticles throughout the plant’s vascular networks increases the risk of exposure to reproductive organs, especially in species with a long flowering period [71]. During the transition from vegetative to reproductive development, changes in floral tissue differentiation can influence pollen viability, ovule development, and fertilization efficiency, thereby determining reproductive success [72].

Multi-omics analyses indicate that the transition from vegetative to reproductive growth is regulated through coordinated changes in transcription factors, chromatin remodeling proteins, and metabolic enzymes. The outcome depends on nanoparticle identity, transformation state, and exposure level [73].

### 3.4. Grain Filling, Maturation, and Product Quality Formation

The last phases of plant growth entail the mobilization of stored resources to seeds, fruit, or storage organs through massive metabolic reconstruction. Long-distance transport redistributes nutrients and may also transport accumulated nanoparticles to developing seeds and fruits [74] [Figure 3]. Nanomaterials accumulated by vegetative tissues can also be co-transported with these assimilates, thereby increasing their presence in edible organs. Subcellular sequestration systems, consisting of vacuolar compartmentalization and cell wall binding, partially restrict nanoparticle movement but do not serve as complete barriers [75]. As a result, trace levels might still be present in commercially harvested products, potentially affecting food safety and nutritional content. Metabolomic studies indicate that exposure to nanoparticles during seed or fruit development may modify storage proteins, lipids, and secondary metabolites, thereby influencing nutritional quality. In some cases, nano-enabled nutrient delivery may also enhance micronutrient accumulation in edible tissues, contributing to biofortification and helping to alleviate hidden hunger [76].

Perturbation during seed germination and early vegetative development can reduce the establishment of sink capacity. In contrast, disturbances during reproductive development, particularly grain filling or fruit maturation, may impair assimilate translocation to developing reproductive organs [77]. Distinguishing the effects of nanomaterial exposure across these developmental stages is essential for identifying stage-specific intervention strategies and predicting their long-term impacts on crop productivity [78].

### 3.5. Towards a Framework of Life-Stage-Specific Vulnerability

Taken together, the results across developmental stages confirm that nanomaterial sensitivity is not evenly distributed over time [79]. Each developmental stage differs in physiology, metabolism, and molecular regulation, leading to different responses to nanomaterial exposure. A combination of omics-based signatures and phenological data allow the development of life-stage-specific vulnerability signatures that can be used to link agronomic performance to molecular perturbations [80]. Such a framework supports predictive modeling of plant–nanomaterial interactions using molecular and phenotypic information [81]. Overall, integrating developmental stage with nanoparticle identity and omics information provides a practical framework for precision nano-agriculture and risk assessment [Figure 3] [Table 1, Table 2, Table 3].

## 4. Cellular and Molecular Uptake Mechanisms

The physicochemical characteristics of nanoparticles govern the uptake of ENMs by plants before they reach biological tissues. Properties such as particle size, morphology, surface charge, and dissolution behavior determine interactions with plant cell walls, plasma membranes, and transport systems [86]. Therefore, nanoparticle uptake depends primarily on nanoparticle physicochemical properties rather than passive diffusion. Understanding these relationships provides the mechanistic link between environmental exposure and the molecular reprogramming revealed by multi-omics analyses [103].

Although significant progress has been made in identifying the principal routes of nanoparticle uptake and translocation in plants, many aspects of their movement within tissues remain poorly understood. Nanoparticles may enter plants through roots or aerial tissues and subsequently move via apoplastic and symplastic pathways before being transported through the xylem and, in some cases, redistributed by the phloem. However, the mechanisms governing nanoparticle movement between tissues and cells remain incompletely understood. These processes differ among engineered nanomaterials such as ZnO NPs, Ag NPs, SiO_2_ NPs, and carbon nanotubes and are further influenced by plant species and environmental conditions. A more comprehensive understanding of cell-to-cell transport and tissue-specific distribution is essential for explaining how nanoparticle localization influences plant physiology, nutrient homeostasis, and stress responses under realistic growing conditions.

### 4.1. Apoplastic and Symplastic Pathways of Nanoparticle Entry

Nanoparticles first interact with external plant surface barriers, including cuticular waxes on leaves, root mucilage, and microbial biofilms associated with the epidermis, before reaching the cell wall–plasma membrane interface, which regulates their entry into the symplastic network [82]. Cell-wall architecture imposes size- and charge-dependent constraints that regulate nanoparticle movement through the apoplast [83]. Cell-wall remodeling during growth and stress further regulates nanoparticle access to the plasma membrane [88].

After crossing the cell wall, nanoparticles enter the symplast, where intracellular transport begins. Plasmodesmatal permeability is dynamically regulated by callose deposition and cytoskeletal remodeling, both of which are influenced by nanomaterial exposure [84]. Some nanoparticles, including carbon nanotubes and graphene oxide, have been reported to alter plasmodesmata permeability through direct or stress-mediated mechanisms [104].

### 4.2. Membrane-Mediated Transport and Endocytic Pathways

Membrane-mediated uptake represents a selective stage of nanomaterial internalization in which nanoparticle physicochemical characteristics largely determine cellular entry. Particle size, surface chemistry, morphology, and surface charge regulate interactions with plasma membrane receptors [94]. Clathrin-mediated endocytosis is considered the principal uptake pathway for many nanoparticles, whereas lipid raft-associated pathways contribute to the uptake of selected nanoparticles depending on their surface properties [95,101].

Following internalization, membrane trafficking coordinates nanoparticle sorting and intracellular distribution while simultaneously influencing cellular signaling processes. Perturbation of membrane organization and transport activity can modify ion homeostasis, vesicular dynamics, and signal transduction pathways, thereby linking nanoparticle uptake with downstream transcriptional and metabolic responses [96,105,106]. Thus, membrane transport links nanoparticle physicochemical properties with downstream molecular responses.

### 4.3. Transport over Vascular Networks over a Long Distance

After entering the cell, the long-distance movement of nanomaterials depends on their physicochemical characteristics, including particle size, surface chemistry, stability, and interactions with endogenous biomolecules, which determine loading into xylem and phloem tissues [87,89]. Small and stable nanoparticles such as ZnO and SiO_2_ nanoparticles may move through the xylem, whereas redistribution through the phloem depends on nanoparticle characteristics and plant species [90,91]. Environmental transformations and interactions with plant metabolites further modify nanoparticle mobility and tissue-specific accumulation, thereby influencing exposure patterns at distant organs [107]. Consequently, vascular transport is a dynamic process that links nanoparticle physicochemical identity to whole-plant distribution and to the spatial regulation of subsequent molecular and physiological responses.

### 4.4. Mechanisms of Subcellular Targeting and Compartmentalization

**Figure 4:**
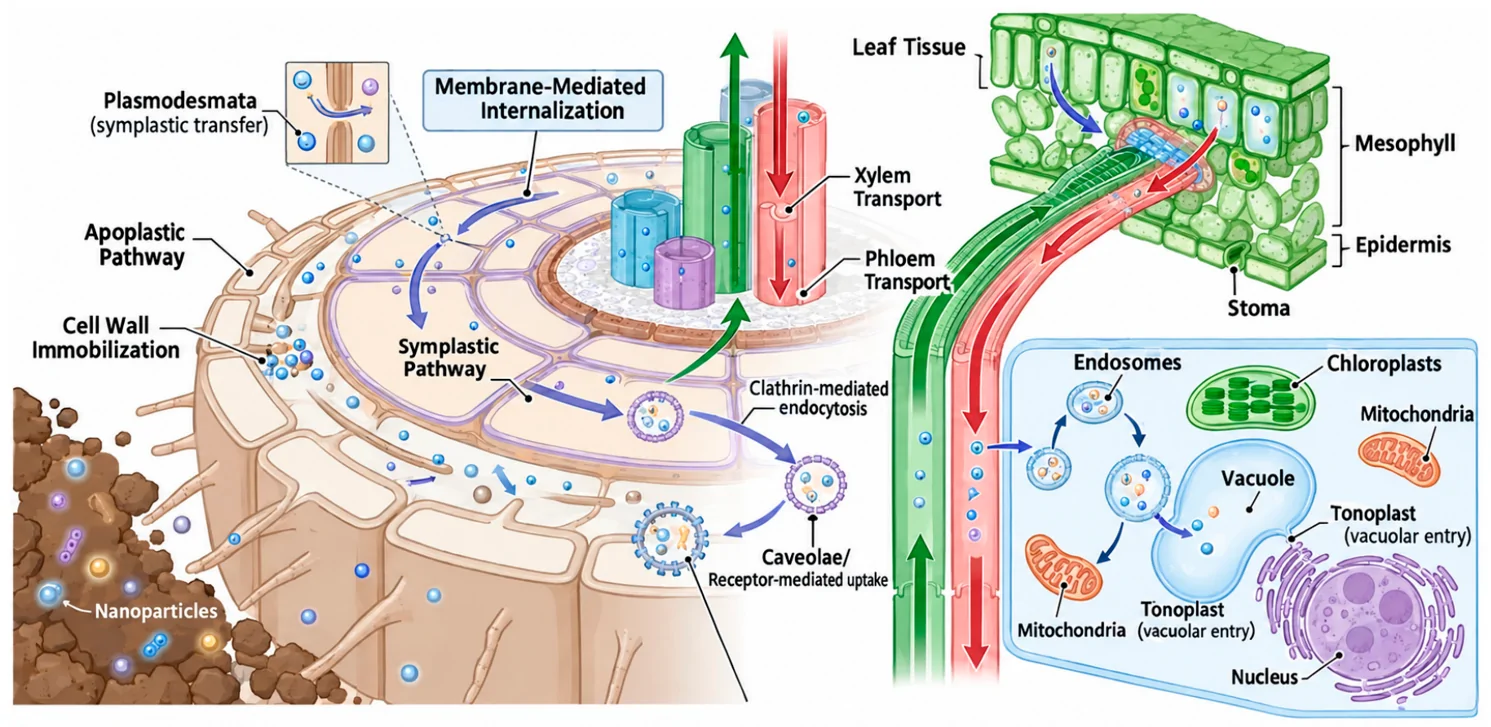
Schematic representation of the cellular and molecular pathways governing nanoparticle uptake, transport, and intracellular targeting in plants. The diagram integrates apoplastic and symplastic entry routes, membrane-mediated internalization, vascular translocation, and subcellular compartmentalization. The grey arrows show the apoplastic transport pathway, while the blue arrows depict the symplastic and membrane-mediated transport pathway. The green arrows point out the xylem transport pathway, and the red arrows illustrate the phloem transport pathway.

Following intracellular transport, nanomaterials are selectively partitioned into distinct subcellular compartments based on their physicochemical characteristics and interactions with cellular trafficking pathways [97]. Their localization within chloroplasts, mitochondria, nuclei, the endoplasmic reticulum, or vacuoles governs the nature and intensity of cellular responses by influencing redox balance, metabolic activity, and signal transduction processes [98,102,108]. Compartment-specific accumulation may either minimize cellular damage through sequestration or enhance biological activity by directly interacting with metabolically active organelles and regulatory networks [109]. Therefore, subcellular targeting is a critical mechanistic step linking nanoparticle physicochemical properties to coordinated transcriptomic, proteomic, epigenetic, and metabolomic reprogramming in plants (Figure 4).

### 4.5. Towards Unified Network Model of Nano-Trafficking

Nanoparticle uptake, intracellular trafficking, and tissue distribution operate as interconnected processes governed by nanoparticle physicochemical characteristics rather than independent biological events [110]. Environmental transformation, membrane interactions, vascular transport, and subcellular localization collectively determine the magnitude and specificity of plant responses by regulating exposure at multiple biological levels [111]. Integrating these transport processes within a unified framework provides a mechanistic basis for understanding how nanoparticle properties influence coordinated transcriptomic, proteomic, epigenetic, and metabolomic reprogramming. Such a systems-level perspective links environmental exposure with molecular regulation and plant performance, establishing the conceptual foundation for the integrative multi-omics analyses discussed in the following sections. Transport proteins, vesicle trafficking components, and plasmodesmata regulate nanoparticle compartmentalization and ultimately influence the molecular responses summarized in Table 1.

## 5. Reprogramming of Transcriptomics and Proteomics

Transcriptomic and proteomic analyses provide mechanistic insight into the early molecular responses of plants following exposure to specific engineered nanomaterials. Changes in gene expression establish the initial regulatory response, whereas alterations in protein abundance, activity, and post-translational modification determine the functional execution of these programs [112].

Molecular responses vary with nanoparticle identity, exposure concentration, and duration and therefore should not be interpreted as uniformly adaptive. Low or moderate concentrations of Ag NPs, SiO_2_ nanoparticles, and TiO_2_ nanoparticles often activate defense-associated pathways. In contrast, prolonged exposure or excessive concentrations, particularly of Ag NPs and metal oxide nanoparticles, promote oxidative damage and disrupt transcriptional and translational regulation [113]. Consequently, transcriptomic and proteomic datasets should always be interpreted in relation to nanoparticle identity and exposure conditions, as the observed molecular responses may reflect either acclimation or toxicity.

### 5.1. Global Remodeling of Gene Expression Landscapes

Transcriptomic studies demonstrate that different engineered nanomaterials induce distinct gene-expression profiles. For example, Ag NPs predominantly regulate oxidative stress- and defense-related genes; ZnO nanoparticles mainly influence metal homeostasis and ion transport pathways; TiO_2_ nanoparticles affect photosynthesis- and carbon metabolism-associated transcripts; whereas SiO_2_ nanoparticles are more frequently associated with drought-responsive and antioxidant gene networks [114]. Ag NP exposure commonly increases the expression of antioxidant genes, including *SOD*, *CAT*, and *APX*, as well as molecular chaperones and glutathione-associated proteins, whereas SiO_2_ nanoparticles preferentially enhance genes involved in osmotic adjustment and dehydration tolerance. These responses contribute to maintenance of redox homeostasis under favorable exposure conditions [115].

Nanoparticle exposure also modifies transporter gene families, although the affected transport systems differ among nanomaterials. ZnO nanoparticles mainly regulate zinc transporters, whereas Ag NPs more frequently influence ABC transporters and vesicle-trafficking components associated with intracellular detoxification. Changes in the expression of membrane transporters, ATP-binding cassette (ABC) transporters, and ion channels alter the intracellular distribution of essential elements and nanoparticles [116]. These transcriptional changes influence nanoparticle uptake, intracellular sequestration, and subcellular compartmentalization, thereby modifying cellular exposure and metal homeostasis. In addition to classical stress responses, nanomaterial-sensitive transcriptomes exhibit reorganization of major metabolic pathways, cell-cycle regulators, and developmental genes [117]. These transcriptional changes indicate that exposure to nanomaterials influences fundamental growth and developmental processes and activates defense responses [118]. Transcriptional responses to nanomaterials are highly context-dependent and often nonlinear, varying with nanoparticle characteristics, environmental conditions, and the plant’s developmental stage. This plasticity indicates the incorporation of nanomaterial-derived cues into existing regulatory hierarchies, but not the activation of a specific pathway, which is nano-specific [119].

### 5.2. Protein Metabolism and Post-Translational Regulatory Dynamics

Proteomic analyses complement transcriptomic data by identifying the functional consequences of altered gene expression through changes in protein abundance, activity, and post-translational regulation [120]. Ag NPs, ZnO nanoparticles, and TiO_2_ nanoparticles alter proteomic profiles by affecting redox balance, energy metabolism, and protein–protein interactions. Redox-sensitive proteins are among the primary targets of nanomaterial-induced proteomic regulation. Depending on the exposure, the enzyme activity and signaling capacity of cysteine residues are reversibly oxidized by reactive oxygen and nitrogen species [121]. These changes occur in essential metabolic enzymes, transcription factors, and scaffold proteins, resulting in rapid adaptation without de novo protein synthesis. Increased abundance of molecular chaperones and unfolded protein response components helps maintain proteome stability during nanomaterial-induced stress [122].

Another major regulatory layer sensitive to nanomaterials is the phosphorylation network. Signal transduction cascades, which interrelate membrane perception and nuclear transcriptional responses, are coordinated by protein kinases and phosphatases. Phosphorylation of proteins involved in hormone signaling, cytoskeletal organization, and vesicle trafficking contributes to nanoparticle uptake and stress adaptation. Ubiquitination, phosphorylation, and SUMOylation can further crosstalk to increase regulatory flexibility, allowing protein regulation at a fine scale [85]. Proteins are selectively degraded through the ubiquitin–proteasome system and autophagy to remove damaged proteins while also regulating developmental processes such as hormone signaling, senescence, and fruit ripening. SiO_2_ nanoparticles have been reported to enhance drought tolerance, whereas Ag NPs primarily induce stress-associated proteome remodeling under controlled exposure conditions. Consequently, coordinated regulation of protein turnover and metabolism plays an important role in determining long-term physiological and phenotypic outcomes [123].

Protein abundance alone does not fully explain plant adaptation to environmental nanomaterials. Reversible post-translational modifications, including phosphorylation, ubiquitination, glycosylation, and redox-dependent modifications, regulate protein activity, stability, intracellular localization, and signaling efficiency. Together, these post-translational mechanisms link transcriptomic responses to functional protein regulation in plants’ responses to engineered nanomaterials.

### 5.3. Recovery of Regulatory Networks and Recovery of Control Nodes

Integrating transcriptomic and proteomic datasets enables the identification of coordinated regulatory modules that control stress perception, cellular signaling, and metabolic adjustment during nanomaterial exposure [124]. Co-expression studies indicate that groups of genes are regulated in a concerted manner across exposure conditions and developmental stages, providing information on functional modules and shared regulatory inputs. These modules commonly include genes associated with redox regulation, hormone signaling, transport systems, and secondary metabolism, demonstrating coordinated molecular responses rather than isolated pathways. Interactome modeling and protein–protein interaction mapping shed additional light on the structural organization of the response networks [125]. Proteins central to hubs are often highly connected and play regulatory roles, making them convergence points for various signaling pathways. Representative regulatory hubs include WRKY and MYB transcription factors, MAPK signaling components, scaffold proteins, and protein kinases that integrate environmental and developmental signals. Perturbation of these regulatory hubs can propagate signaling across interconnected pathways, thereby influencing multiple physiological processes simultaneously [126].

Multi-omics analyses have identified key regulators that integrate transcriptional and post-translational responses. These regulators coordinate chromatin accessibility, hormone signaling, and metabolic activity, thereby shaping the overall molecular response to nanomaterial exposure. Their activity is dynamically controlled through phosphorylation, redox regulation, and protein–protein interactions, enabling rapid adjustment to changing nanomaterial exposure conditions [99].

### 5.4. The Systems-Level Regulatory Hubs and Adaptive Reprogramming

Integrated transcriptomic and proteomic analyses identify regulatory hubs that coordinate gene expression with protein activity during nanomaterial exposure [127]. These hubs integrate redox, hormonal, and transport-related signals, enabling coordinated regulation of stress-responsive pathways and resource allocation [128]. Identification of these regulatory hubs improves our understanding of how plants coordinate molecular responses to different engineered nanomaterials under diverse environmental conditions [129]. Table 4 summarizes the principal transcriptomic and proteomic responses reported for representative engineered nanomaterials.

Although transcriptomic and proteomic analyses provide valuable insights into early molecular responses following nanomaterial exposure, these datasets alone cannot fully explain the complexity of plant adaptation. Their biological significance becomes clearer when integrated with epigenetic and metabolomic data, providing a more comprehensive understanding of stress perception, signaling, and physiological adaptation. Therefore, an integrated multi-omics perspective is essential for identifying the regulatory networks that underpin plant responses to ENMs.

## 6. Epigenetic Reprogramming and Memory of Generations

ENMs modify epigenetic regulatory mechanisms that influence gene expression beyond the immediate exposure period. Alterations in DNA methylation, histone modifications, chromatin organization, and small RNA pathways contribute to transcriptional plasticity and, in some cases, generate heritable molecular changes that influence plant performance across developmental stages and subsequent generations [92,140]. Understanding nanoparticle-specific epigenetic regulation is essential for predicting delayed, cumulative, and transgenerational effects in plants.

### 6.1. Epigenomic Plasticity and the Dynamics of DNA Methylation

DNA methylation is a key process of maintaining genome stability and gene expression in plants. It is also dynamic and operates across a variety of sequential contexts in response to environmental cues [133]. Ag NPs, ZnO nanoparticles, and TiO_2_ nanoparticles have each been reported to modify DNA methylation at different genomic regions, indicating nanoparticle-specific epigenetic regulation. These locus-specific changes alter transcriptional accessibility and affect the subsequent responsiveness of the target genes to the stimulus [141]. Both hypermethylation and hypomethylation have been reported, depending on nanoparticle properties and exposure conditions. Methylation amplification of promoter regions can inhibit the expression of growth-related genes under stress, and demethylation of defense-related loci can promote rapid activation of defense systems [134]. Under prolonged exposure, some nanomaterials may induce stable epimutations that persist beyond the initial stress period. These epimutations introduce heritable variability in regulatory networks, which can have developmental consequences and influence stress responses [142].

These methylation patterns are more complex to establish and maintain through interactions among DNA methyltransferases, demethylases, and chromatin-associated proteins. Overall, DNA methylation responses differ among nanoparticle classes and should therefore be interpreted within the context of nanoparticle identity and exposure conditions [135].

### 6.2. Processes of Histone Modifications and Remodeling of Chromatin Architecture

**Figure 5:**
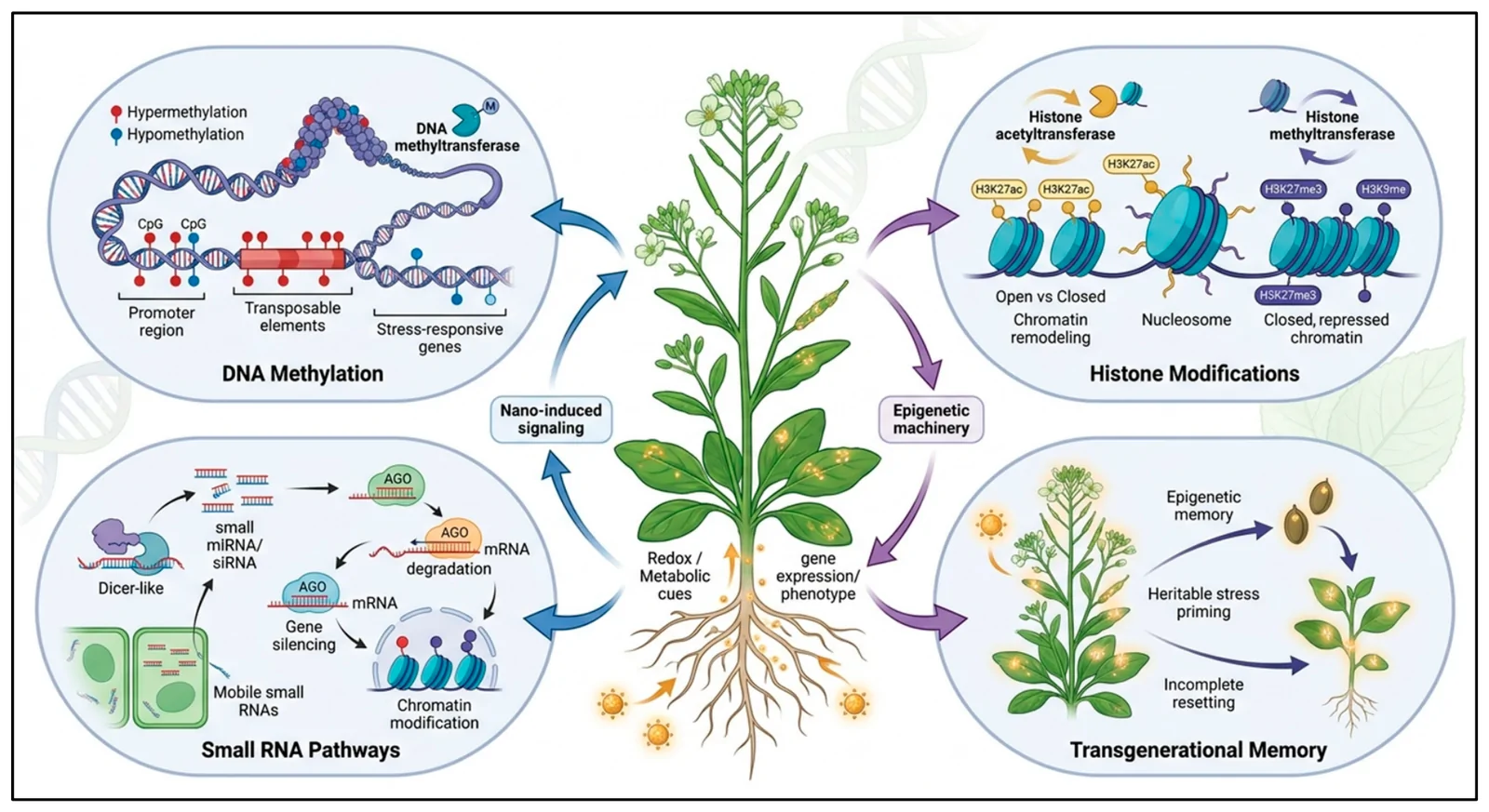
Schematic representation of nano-induced epigenetic reprogramming in plants, highlighting DNA methylation, histone modifications, small RNA pathways, and transgenerational memory effects. The arrows point to the direction of interactions between nanotechnology-induced signaling and their respective epigenetic processes. Blue and purple arrows denote the relationships between DNA methylation/small RNA pathways and histone modifications/transgenerational memory, respectively, while the internal arrows show the process flow in each pathway.

Histone modifications provide an additional regulatory layer that determines chromatin accessibility and transcriptional activity during nanomaterial exposure [143]. TiO_2_, Ag, and carbon-based nanomaterials have been reported to alter histone modification patterns, although the affected regulatory pathways differ among nanoparticle classes. Changes in the patterns of trimethylation and acetylation at the major lysine residues affect nucleosome stability and transcription factor accessibility [136]. For instance, enrichment of activating marks around regulatory regions could sustain the expression of detoxification and signaling genes. In contrast, accumulation of repressive marks could limit growth-related pathways during prolonged stress. The histone-modifying enzymes that mediate these modifications are regulated by cellular energy and redox conditions [144] [Figure 5].

Alterations in histone modification patterns are often coupled with massive chromatin remodeling events that change the three-dimensional organization of the genome. Increased chromatin access to specific genomic regions leads to rapid transcriptional responses, whereas compaction of other regions limits unnecessary metabolic expenditure. Such chromatin reorganization enables selective activation of adaptive pathways while restricting unnecessary transcription during nanoparticle exposure [100].

### 6.3. Small RNA-Mediated Regulatory Networks

MicroRNAs and small interfering RNAs refine gene expression by regulating transcript stability and RNA-directed DNA methylation during nanomaterial exposure [137]. Ag NPs, ZnO nanoparticles, and carbon-based nanomaterials have been associated with distinct changes in small RNA expression, indicating nanoparticle-specific regulatory responses [145]. Small interfering RNAs are involved in maintaining silencing at transposable elements and repetitive regions, and they provide genome integrity during times of stress. In other instances, nanomaterial-responsive small RNAs serve as mobile cues, whereby control information is communicated between tissues and developmental stages [93]. The complex interplay between the small RNA pathways and the DNA methylation and histone modification programs enables the coordination of gene expression at multiple levels. The effect of perturbing any component can be amplified or nullified and spread throughout the epigenetic network [146].

### 6.4. Transgenerational Transmission and the Formation of Epigenetic Memory

Persistent epigenetic changes have been reported for selected nanoparticle classes, although evidence for transgenerational inheritance remains limited [147,148]. The persistence of epigenetic memory depends on nanoparticle identity, exposure level, developmental stage, and plant genotype.

The maintenance of epigenetic memory involves incomplete resetting of methylation patterns, stable histone marks, and hereditary small RNA populations. These processes allow plants to incorporate short-term environmental dynamics into long-term regulatory strategies and make the impact of nanomaterial exposure more than that of individual life cycles [149].

### 6.5. Epigenetic Memory Conceptualization of Nano-Induced

Emerging evidence indicates that certain engineered nanomaterials can act as epigenetic modulators, although responses remain nanoparticle-specific. Plants transform physicochemical exposure into long-term molecular information through coordinated alterations in DNA methylation, histone structure, and small RNA networks [150]. Developmental plasticity, stress resilience, and generational stability are affected by this nano-induced epigenetic memory. Environmental signals within chromatin structures allow plants to archive past conditions as a molecular memory that directs future responses. Integrating epigenomics with transcriptomics, proteomics, metabolomics, and phenotypic analyses will improve understanding of long-term nanoparticle responses [151]. These insights can serve as a basis for forecasting long-term nano-environmental effects and crafting strategies to advance sustainable initiatives that bridge technological change and biological flexibility [Figure 5].

Epigenetic regulation extends beyond DNA methylation to include histone acetylation, methylation, chromatin remodeling, and regulatory small RNAs. Together, these mechanisms influence chromatin accessibility and gene expression, enabling plants to coordinate developmental processes and environmental adaptation following exposure to nanomaterials.

## 7. Metabolomic Remodeling and Systems-Level Modeling of Plant–Nanomaterial Interactions

Metabolic networks represent the functional outcome of coordinated transcriptomic, proteomic, and epigenetic regulation in plants [130]. In turn, changes in metabolite composition provide a direct readout of the translation of nanomaterial exposure into physiological and agronomic effects. Different engineered nanomaterials alter primary and specialized metabolic pathways through distinct molecular mechanisms rather than producing a common metabolic response. Metabolomic profiling, in combination with integrative data analysis and computational modeling, can be used to recapitulate system-wide response architectures that connect molecular perturbations to phenotypic expression [152]. Ag NPs, TiO_2_ nanoparticles, ZnO nanoparticles, and SiO_2_ nanoparticles have been reported to alter carbon metabolism, nitrogen utilization, and energy production, although the affected pathways differ among nanoparticle classes. Adjustments in photosynthetic efficiency, respiratory rate, and carbohydrate partitioning affect the proportions between growth and maintenance processes [153]. Adaptive resource reprogramming is indicated by changes in fixed carbon toward defense compounds or stress-mitigation systems [153]. On the same note, nitrogen metabolism may be altered, which affects amino acid synthesis, protein breakdown, and the formation of signaling molecules that influence structural growth and regulation. Changes in mitochondrial and chloroplastic energy metabolism also alter ATP supply and redox homeostasis, amplifying feedback controls between metabolic conditions and stress perception.

In addition to primary metabolism, nanomaterials strongly impact secondary specialized metabolite biosynthesis. These compounds are mainly important in antioxidative defense, signaling, and ecological interactions. Ag NPs and ZnO nanoparticles frequently increase phenolic and flavonoid accumulation through activation of the phenylpropanoid pathway under controlled exposure conditions [131]. Alkaloid production can be regulated by varying precursor supply and controlling enzymes that affect the ability to defend against biotic stress. Terpenoid and glucosinolate pathways are also similar in their plasticity and respond to perturbations in precursor fluxes and transcriptional regulation induced by nanoperturbations. These metabolic adaptations not only enhance stress tolerance but also transform the relationships among plants, pollinators, herbivores, and microbes.

The process of metabolic remodeling is tightly linked to the dynamics of phytohormones, which are key to integrating developmental and environmental signals. TiO_2_ nanoparticles, SiO_2_ nanoparticles, and ZnO nanoparticles have been reported to modify hormone biosynthesis and signaling, although responses vary with nanoparticle identity and exposure conditions [138]. Root and shoot architecture are affected by an altered auxin gradient, and abscisic acid signaling is altered, thereby inducing stomatal regulation and stress acclimation. Alterations in ethylene and jasmonate signaling regulate senescence, defense mechanisms, and reproductive development. The interaction of these hormonal networks orchestrates metabolism in relation to morphological and physiological responses and to context-dependent adaptation to the outside world.

Bioactive metabolites are among the most sensitive indicators of plant responses to environmental nanomaterials, reflecting how molecular changes are translated into functional biochemical adaptations [132]. Unlike primary metabolites that support growth, secondary metabolites contribute to antioxidant defense, stress acclimation, signaling, and ecological interactions. Different nanoparticle classes regulate the accumulation of phenolics, flavonoids, alkaloids, terpenoids, glucosinolates, and carotenoids through distinct biosynthetic pathways [26]. These metabolic changes depend on nanoparticle characteristics, exposure concentration, plant species, and developmental stage. Controlled exposure may enhance antioxidant capacity, nutritional quality, and the accumulation of pharmacologically important compounds, whereas excessive exposure can disrupt metabolic homeostasis and suppress metabolite biosynthesis. Consequently, metabolite profiles should be interpreted along with transcriptomic, proteomic, and epigenetic datasets to elucidate nanoparticle-specific metabolic responses [139].

Metabolic reprogramming similarly reflects a balance between adaptation and toxicity. The biological outcome depends on nanoparticle identity, exposure concentration, plant species, and developmental stage, resulting in either metabolic acclimation or metabolic disruption.

The reorganization of the metabolic and hormonal systems has direct implications for the nutritional and pharmacological quality of plant-derived products. Changes in exposure to amino acids, vitamins, antioxidants, and specialized metabolites control the functional food properties and health-related features [154]. Increased bioactivity levels can enhance nutraceutical value, and their alteration can negatively affect nutritional balance. These effects help determine food safety, consumer health, and the use of nanomaterials in future targeted metabolite enhancement strategies. High-throughput multi-omics approaches have considerably improved the understanding of plant responses to ENMs by enabling simultaneous analysis of interconnected molecular processes. Rather than functioning independently, epigenetic modifications regulate chromatin accessibility and transcriptional activity, thereby influencing the expression of genes involved in nanoparticle uptake, oxidative stress responses, hormone signaling, and metabolic regulation. The resulting transcriptional changes alter protein abundance and activity through post-translational regulation, ultimately redirecting metabolic pathways responsible for carbon allocation, redox homeostasis, and secondary metabolite biosynthesis. Integrative network analysis links transcriptomic, proteomic, epigenetic, and metabolomic datasets to identify nanoparticle-specific regulatory modules associated with plant adaptation [155].

Machine-learning approaches have become valuable tools for integrating transcriptomic, epigenomic, proteomic, and metabolomic datasets generated from plants exposed to ENMs. By combining molecular profiles with nanoparticle physicochemical characteristics and phenotypic observations, these approaches can identify regulatory modules associated with nanoparticle uptake, oxidative stress responses, hormone signaling, nutrient metabolism, and secondary metabolite biosynthesis. Such integrative analyses facilitate the identification of key molecular biomarkers, reveal coordinated regulatory pathways across multiple omics layers, and improve predictions of plant responses under different environmental exposure conditions. Machine-learning models therefore improve interpretation of complex multi-omics datasets while supporting nanoparticle-specific prediction of plant responses [156].

**Figure 6:**
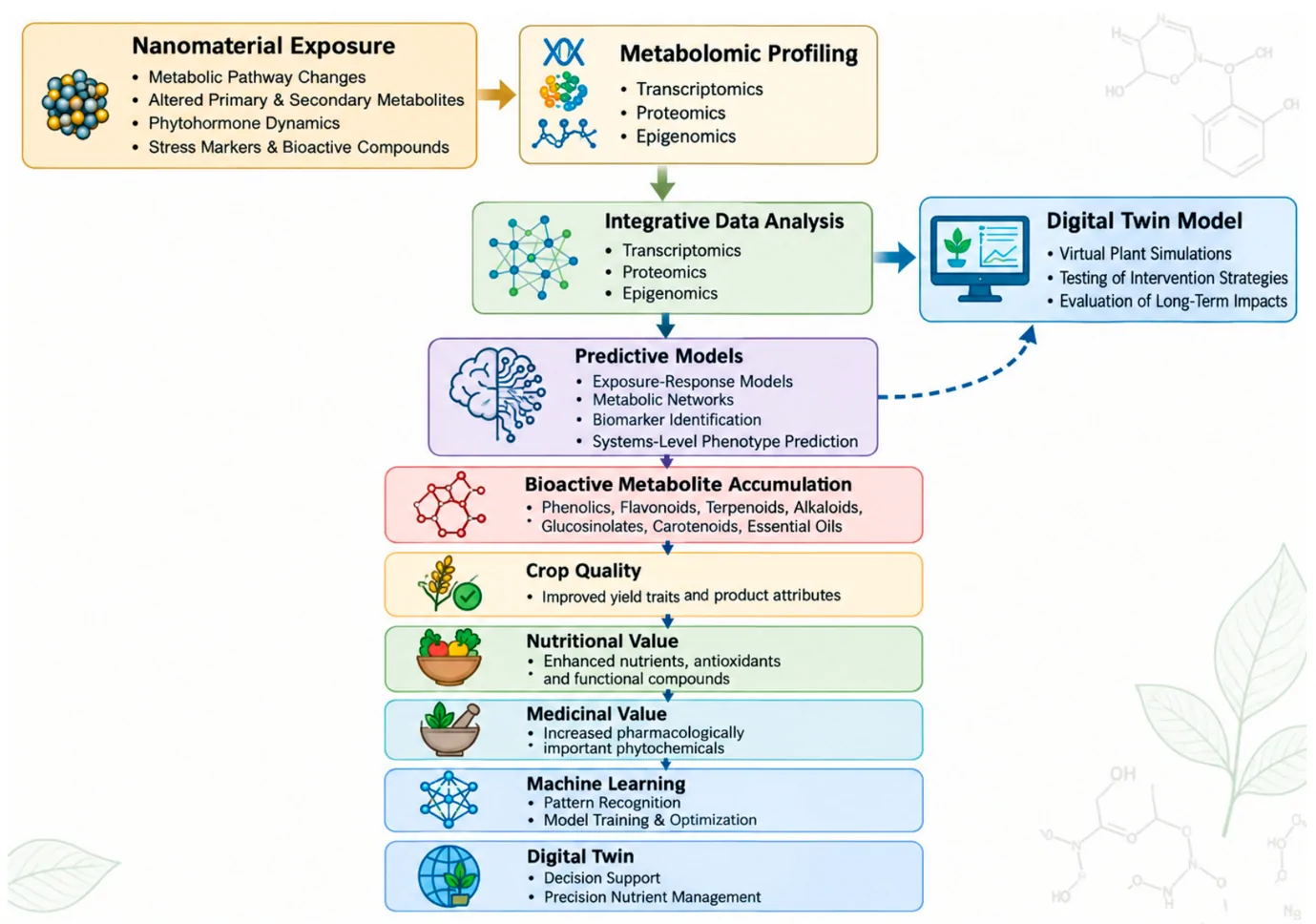
Systems-level framework illustrating how environmental nanomaterial exposure drives coordinated multi-omics regulation, leading to bioactive metabolite accumulation, predictive modeling, and digital twin development for improving crop quality, nutritional value, and medicinal potential.

Digital twin technology extends multi-omics integration by generating virtual representations of plant–nanomaterial systems that combine molecular, physiological, and environmental information within a single predictive framework. These models incorporate nanoparticle physicochemical properties along with transcriptomic, epigenomic, proteomic, metabolomic, and phenotypic data to simulate plant growth, stress adaptation, nutrient utilization, and metabolic reprogramming across different exposure scenarios. By continuously integrating experimental observations with computational predictions, plant-specific digital twins can support mechanistic interpretation of nanomaterial behavior, optimize exposure strategies, and facilitate the rational design of environmentally sustainable nano-enabled agricultural technologies [156]. These models integrate molecular networks, nanoparticle transport, and environmental variables to simulate nanoparticle-specific plant responses under different exposure scenarios (Figure 6).

Integrating multiple omics datasets enables the construction of regulatory networks that identify key molecular interactions linking nanoparticle exposure with plant responses. Network-based analyses facilitate the identification of regulatory hubs, while digital twin frameworks use these integrated datasets to simulate plant performance under different environmental conditions. Together, these approaches provide a systems-level framework for predicting nanoparticle-specific plant responses while supporting the development of safe and sustainable nano-enabled agriculture.

## 8. Effects of Nanomaterials on Yield Formation and Soil–Plant–Microbiome Continuum

Crop productivity represents the cumulative outcome of molecular regulation, physiological adjustment, and environmental interactions occurring throughout plant development. Consequently, evaluating yield formation together with plant–soil–microbiome interactions provide the functional context for interpreting multi-omics responses to different engineered nanomaterials [157]. A combination of yield-related processes and rhizosphere-level dynamics is thus a comprehensive approach to assessing the functional outcomes of nanomaterial exposure.

### 8.1. Source–Sink Reorganization and Dynamics of Carbon Allocation

The formation of the yield is based on the effective coordination of photosynthetic source tissues and developing sink organs. Ag NPs, TiO_2_ nanoparticles, ZnO nanoparticles, and SiO_2_ nanoparticles have been reported to alter photosynthetic capacity, stomatal regulation, and nutrient assimilation, although the magnitude of these effects differs among nanoparticle classes. Altered carbohydrate synthesis and transport affect phloem loading efficiency and long-distance allocation patterns, reorganizing sink strength in roots, reproductive organs, and storage tissues [158].

Transcriptomic and metabolomic data show that exposure usually results in reprogramming of enzymes that mediate sucrose metabolism, starch turnover, and glycolytic pathways. In some cases, increased carbon allocation to defense and detoxification processes may limit the resources available for vegetative growth and reproductive development. Conversely, improved nutrient use efficiency and photosynthetic performance can enhance biomass accumulation, strengthen sink activity, and support both plant growth and reproductive success, depending on the nanomaterial properties and environmental conditions [159]. These outcomes depend on nanoparticle identity, exposure concentration, developmental stage, and environmental conditions.

### 8.2. Reproductive Development, Fertility and Stable Yield

Reproductive success is a key factor in determining yield stability and is highly sensitive to environmental perturbations. Ag NPs, ZnO nanoparticles, and metal oxide nanoparticles have been reported to affect reproductive development when exposed during floral initiation, gametogenesis, or fertilization [160]. These effects can alter pollen development, stigma receptivity, and ovule viability, thereby altering fertilization effectiveness. Proteomic and epigenetic analyses indicate that reproductive tissues exhibit distinct molecular stress responses. Changes in mitochondrial performance and antioxidant activity in pollen grains or developing embryos could impair energy supply and genomic integrity. High levels of nanomaterial exposure may reduce pollen viability, impair pollen tube growth, or disrupt embryogenesis, leading to decreased seed set and increased reproductive failure. In contrast, low or appropriately optimized doses can stimulate adaptive responses that enhance stress tolerance and support reproductive development, illustrating the dose-dependent nature of plant responses to nanomaterials [161]. These contrasting responses further demonstrate that reproductive outcomes are nanoparticle- and dose-dependent.

### 8.3. Relations Between Quality and Quantity and Nutritional Outcomes

The definition of crop productivity can be applied in terms of yield quantity and the nutritional and functional quality of the products. Nanoparticle-induced metabolic remodeling influences the accumulation of proteins, carbohydrates, lipids, vitamins, and specialized metabolites [162]. Changes in storage compound accumulation may improve nutritional quality while reducing total biomass. Metabolomic studies indicate that alterations in amino acid and micronutrient composition are frequently accompanied by changes in the accumulation of antioxidants and secondary metabolites. These alterations can enhance the properties of functional foods and cause variability in processing and storage characteristics. From an agronomic perspective, maximizing the benefits of nanomaterial application requires predictive frameworks that integrate exposure conditions with soil characteristics, plant species and genotype, thereby enabling context-specific management strategies that improve productivity while minimizing environmental risks [163]. The accumulation of trace nanoparticles in edible tissues, along with changes in metabolic profiles, necessitates a rigorous evaluation of nanoparticles effects on consumer health and the development of regulatory limitations.

### 8.4. Controlled Environment and Field Systems Evidence

Most of mechanistic understanding of interactions between plants and nanomaterials has been derived from controlled experiments conducted under simplified conditions. Although valuable for mechanistic studies, controlled experiments do not fully represent field conditions [164]. Field studies indicate that nanoparticle behavior differs substantially from controlled laboratory conditions because soil properties, climate, microbial communities, and agricultural practices modify nanoparticle fate. Under field conditions, nanomaterials undergo continuous physicochemical transformations, including aggregation, dissolution, surface modification, and interactions with soil organic matter and mineral surfaces. These transformations alter nanoparticle mobility and bioavailability depending on soil conditions. Furthermore, plants are simultaneously exposed to multiple stressors, such as drought, nutrient limitation, and pathogen pressure, which interact with nanomaterial-induced responses and generate complex outcomes that cannot be predicted from single-factor laboratory experiments [165]. Addressing these knowledge gaps will require coordinated, multi-location field trials conducted across contrasting agroecological regions, together with long-term monitoring and the integration of multi-omics datasets with agronomic performance indicators.

### 8.5. Rhizosphere Engineering and Restructuring of Microbial Communities

Ag NPs, TiO_2_ nanoparticles, ZnO nanoparticles, CeO_2_ nanoparticles, and carbon-based nanomaterials have been reported to alter rhizosphere structure through distinct physicochemical interactions with soil and root surfaces. High-throughput sequencing studies have shown that these changes may alter the relative abundance of beneficial microorganisms, including *Pseudomonas*, *Bacillus*, and *Rhizobium*, and affect taxa involved in nitrogen cycling and stress adaptation. The magnitude and direction of these shifts depend on nanomaterial type, concentration, soil characteristics, and plant species [166]. Such microbial changes affect nutrient mobilization, phytohormone production, and pathogen inhibition, thereby indirectly affecting plant productivity. Beneficial microbial enrichment may improve nutrient acquisition, whereas disruption of mutualistic interactions can reduce plant productivity. The magnitude of these responses depends on nanoparticle properties, soil characteristics, and plant species.

### 8.6. Functional Implications of Nanoparticle-Induced Microbiome Shifts

While numerous studies have demonstrated that nanoparticle (NP) exposure alters the taxonomic composition and diversity of rhizosphere microbial communities, these observations alone provide limited insight into the biological consequences for plants. The rhizosphere microbiome performs essential functions, including nutrient cycling, nitrogen fixation, phosphorus solubilization, phytohormone production, pathogen suppression, and regulation of plant stress responses. Therefore, NP-induced shifts in microbial community structure should be interpreted in terms of their functional implications rather than taxonomic changes alone. Recent studies increasingly employ predictive bioinformatic approaches, such as functional gene inference from microbial community profiles, to estimate potential metabolic alterations; however, these predictions require experimental validation to establish causal relationships between changes in microbial functional and plant performance. Furthermore, the effects of NPs cannot be generalized across all nanomaterials. Metallic nanoparticles (e.g., Ag and ZnO), metal oxide nanoparticles (e.g., TiO_2_ and CeO_2_), carbon-based nanomaterials, and polymeric nanoparticles possess distinct physicochemical properties, dissolution behavior, and reactivity that influence their interactions with soil microorganisms differently. Likewise, soil characteristics, including pH, texture, organic matter content, moisture, and the indigenous microbial community, strongly influence NP transformation and bioavailability, leading to location-specific responses. Future research should therefore integrate microbial community composition with functional analyses, plant physiological measurements, and soil characteristics to establish mechanistic links between NP exposure, microbiome function, and plant health under realistic environmental conditions.

### 8.7. Modulation of Plants-Microbe Communication Networks

Plant–microbe interactions are regulated by complex signaling networks involving metabolites released in root exudates, microbial metabolites, volatile organic compounds (VOCs), and quorum-sensing molecules, which together coordinate microbial colonization, nutrient exchange, and plant stress responses [167]. Different nanoparticle classes can interfere with plant–microbe communication through distinct interactions with signaling molecules and receptor-mediated pathways. These interactions may influence quorum-sensing pathways and the exchange of chemical signals between plants and associated microorganisms, thereby affecting biofilm formation, root colonization, symbiotic associations such as rhizobial nodulation and mycorrhizal establishment, and the expression of microbial virulence factors. The magnitude and direction of these responses depend on nanomaterial composition, concentration, surface properties, and the physicochemical characteristics of the surrounding environment [168]. At the plant level, changes in signaling affect immune responses, nutrient uptake strategies, and developmental plans. These signaling responses are closely integrated with transcriptomic, proteomic, and metabolomic regulation, reinforcing system-level adaptation.

### 8.8. Tripartite Implications of Adaptation and Translation

**Figure 7:**
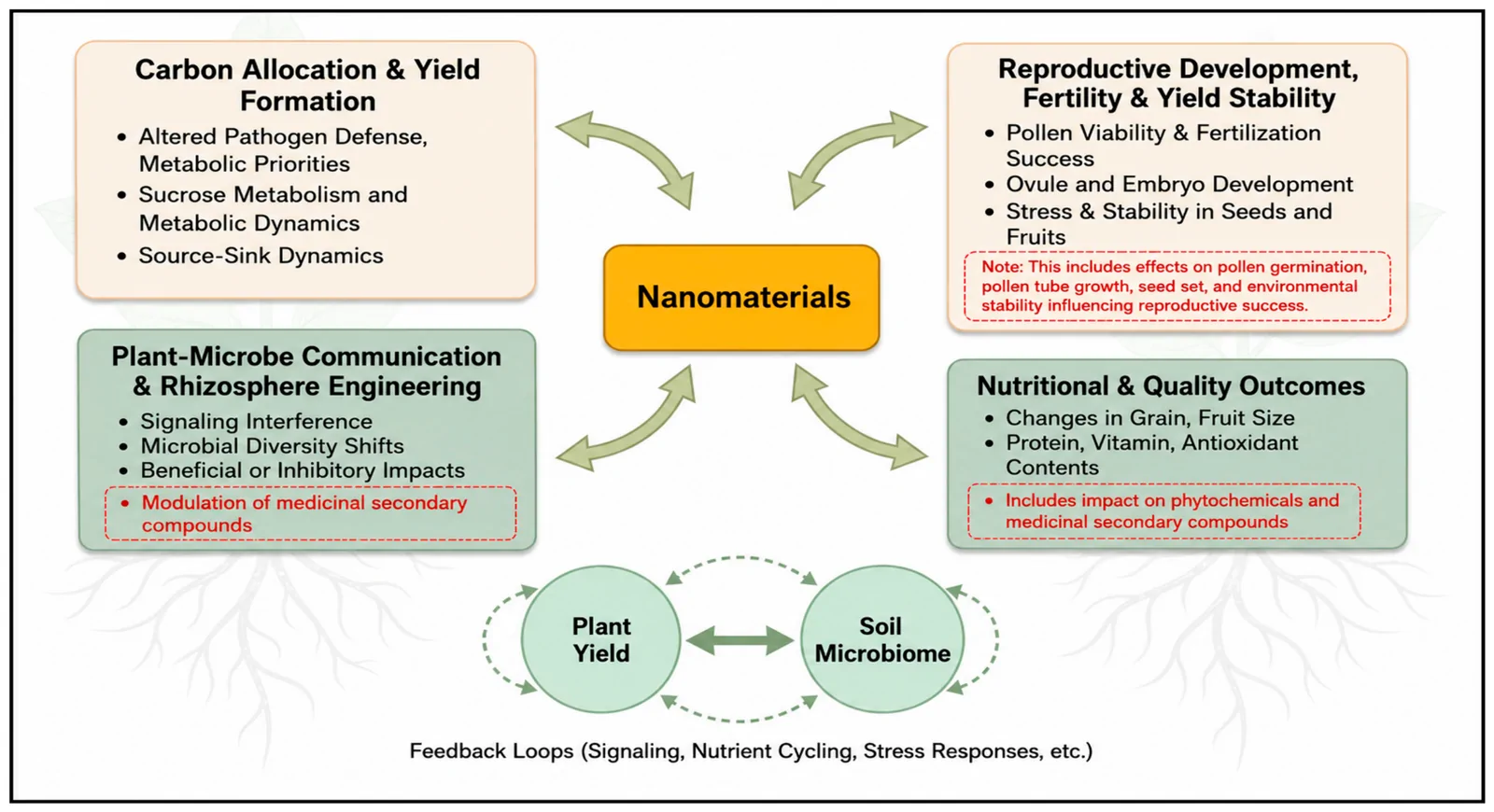
Conceptual framework illustrating how nanomaterials influence plant yield through interconnected effects on carbon allocation, reproductive development, soil microbiome dynamics, and quality traits, mediated by continuous soil–plant–microbe feedback loops. The solid arrows show how the nanomaterials affect the physiological and functional performance of plants, while the double arrows show the two-way interactions between plant productivity and the soil microorganisms. The dotted arrows show feedback mechanisms.

Individual and collective relationships among plants, nanomaterials, and microbial communities establish dynamic networks that define the resilience and sustainability of agricultural ecosystems. Coordinated regulation at the molecular, organismal, and community levels results in adaptive responses [169]. Omics technologies can provide information on metabolic status, hormonal regulation, and microbial community composition that may help identify biomarkers associated with crop performance and stress tolerance. However, translating these molecular signatures into practical agronomic decision-making remains challenging and requires validation across diverse soils, climates, and cropping systems. Future progress will depend on integrating molecular, environmental, and agronomic datasets with field-based evidence to develop management strategies that are scientifically robust, economically feasible, and environmentally sustainable [170]. Together, these interactions provide a systems-level framework for understanding nanoparticle-specific effects on sustainable agricultural productivity [Figure 7].

### 8.9. Dose-Dependent Effects of Green-Synthesized Nanoparticles on Phytotoxicity, Plant Growth, and Yield

Phytotoxicity in plants exposed to green-synthesized nanoparticles is largely dose-dependent and is also influenced by particle size, shape, and the plant species under study [171,172]. Silver nanoparticles (Ag NPs) show size- and concentration-dependent toxicity in terrestrial plants, with exposure decreasing seed germination and inhibiting seedling growth, particularly affecting the mass and length of roots and shoots. Smaller Ag NPs penetrate plant tissues more readily and release silver ions more easily, which makes them more phytotoxic than larger particles at the same concentration [173]. Therefore, particle size and concentration should be evaluated together when assessing the safety of nanomaterial formulations, as both parameters influence their biological effects and environmental behavior [171,174].

Although this review primarily focuses on environmental nanomaterials released into terrestrial ecosystems, green-synthesized nanoparticles are briefly discussed because they represent an emerging class of engineered nanomaterials designed to reduce environmental risks while maintaining agricultural functionality. Their inclusion is intended to provide a comparative perspective on how nanoparticle synthesis strategies may influence plant responses, environmental behavior, and future sustainable applications rather than to shift the primary focus of this review.

Several green-synthesized nanoparticles stimulate plant growth at low to moderate concentrations, whereas higher concentrations frequently induce phytotoxicity [171,172,175]. Synthesized zinc oxide nanoparticles (ZnO NPs) from onion peel waste and observed that their effect on mung bean and wheat shifted from beneficial to phytotoxic as the applied concentration increased, with high doses leading to chlorosis and stunted growth due to Zn excess [172,175]. These findings illustrate the characteristic hormetic response observed for several green-synthesized metal and metal oxide nanoparticles [171,172,175].

The mechanism behind this threshold effect is mostly attributed to reactive oxygen species (ROS) [171,176]. In one study, ZnO nanoparticles synthesized using *Coleus forskohlii* leaf extract were applied as a foliar spray to drought-stressed tomato plants. Concentrations of 25 and 50 mg/L increased shoot and root biomass while reducing malondialdehyde and hydrogen peroxide levels, whereas 100 mg/L increased oxidative stress and diminished these beneficial effects. Similar dose-dependent responses have also been reported for conventionally synthesized ZnO nanoparticles, indicating that the synthesis route alone does not determine biological performance [171,175]. These findings demonstrate that the same nanoparticle formulation may function either as a biostimulant or a stressor, depending on the exposure concentration and plant species [176].

Beyond oxidative stress, accumulated metal ions from nanoparticle dissolution can also disturb nutrient balance and yield components [171,172,177]. High concentrations of Zn or Ag taken up through roots or leaves can reduce chlorophyll content, photosynthetic efficiency, and, under severe exposure, grain or fruit yield. Conversely, low-dose exposure may stimulate beneficial physiological responses consistent with hormetic regulation [18,171,172]. Consequently, yield responses reported for green-synthesized nanoparticles range from significant improvement to yield reduction. This variability is influenced not by the green synthesis route alone but by the interaction among nanoparticle properties, application dose and exposure method, plant species and genotype, soil characteristics, environmental conditions, and the composition of the plant-associated microbiome, all of which collectively determine plant responses [171,172,173,177] (Table 5).

Collectively, these findings demonstrate that plant responses to green-synthesized nanoparticles are governed by nanoparticle properties, exposure dose, and plant characteristics rather than by the synthesis route alone [171,172,173,175]. Further studies are needed to establish standardized exposure concentrations across plant species and experimental systems to define safe and effective application ranges for green-synthesized nanomaterials. Equally important is the development of a clear and consistent definition of green synthesis, together with standardized reporting criteria, to improve comparisons among studies and facilitate the interpretation of biological responses [171,172,177].

## 9. Regulatory Implications, Food Safety, and Ecological

The increasing use of engineered nanomaterials (ENMs) in industry and agriculture has expanded their release into terrestrial and aquatic ecosystems, raising concerns about their long-term environmental and food safety implications. Although plants represent important entry points for engineered nanomaterials into agricultural food chains, different nanoparticle classes vary considerably in their environmental behavior, persistence, and trophic transfer potential. Consequently, their environmental effects should be considered within interconnected soil and aquatic food webs rather than at the level of individual organisms alone [178]. Understanding nanoparticle-induced molecular and physiological changes across food webs is therefore essential for developing science-based regulatory frameworks. Nanomaterials deposited in plant tissues could be passed to subsequent trophic levels via herbivory and dietary ingestion, creating pathways for biomagnification within ecosystem networks. Nanoparticles and metabolites can be absorbed by insects, soil invertebrates, and grazing animals that feed on exposed plants, thereby altering metabolic and reproductive processes [172]. These impacts can affect population dynamics, species interactions, and ecosystem services, including pollination and nutrient cycling. The plant–insect–human continuum is one of the most significant exposure pathways in agroecosystems, since nanoparticles or nano-related metabolic products can enter the human diet through cereals, fruits, and vegetables, as well as animal products. Transformation during food processing and digestion further complicates exposure assessment, emphasizing the need for integrated food-chain evaluation [179].

Conventional risk assessment systems have been based on concentration-based toxicity levels and short-term exposure measures. Although important, these methods do not typically capture sublethal, cumulative, and transgenerational effects, as demonstrated by molecular profiling [180]. High-throughput omics technologies provide sensitive molecular indicators that complement conventional toxicity assessments. Early-warning indicators of physiological perturbation prior to the onset of visible injury include transcriptomic, proteomic, epigenetic, and metabolomic signatures of nanomaterial exposure. These biomarkers improve detection of chronic stress and early molecular perturbations before visible symptoms appear.

The incorporation of omics-derived indicators into risk assessment models improves predictive ability by linking exposure levels to specific regulatory and metabolic pathways [181]. Systems-level analyses identify regulatory modules and biological pathways associated with nanoparticle exposure across different environmental conditions. This information enables extrapolation of laboratory experiments to natural environments and assists in creating stratified assessment strategies with greater emphasis on high-risk situations. Moreover, molecular signatures can be used to separate the effects of natural nanoparticle backgrounds from anthropogenic pollution and to enhance monitoring quality. Irrespective of these developments, major issues remain regarding the transfer of scientific knowledge into regulatory action [182]. The existing policy frameworks tend not to be standardized across nanomaterial definitions, exposure metrics, and testing methodologies. Variations in material synthesis, surface modification, and environmental transformation make it more difficult to develop universal exposure thresholds. Inconsistent experimental protocols and reporting standards further limit data comparability and regulatory harmonization [183].

Guidelines for testing are often based on bulk-material models and are not necessarily representative of nano-specific behavior, e.g., aggregation dynamics, corona formation, and life-cycle transformation. Current regulatory assessments also emphasize acute toxicity while providing limited consideration of chronic, developmental, and transgenerational responses identified through multi-omics studies [184]. Addressing these limitations requires standardized nanoparticle characterization, environmentally relevant exposure models, and long-term monitoring strategies.

**Figure 8:**
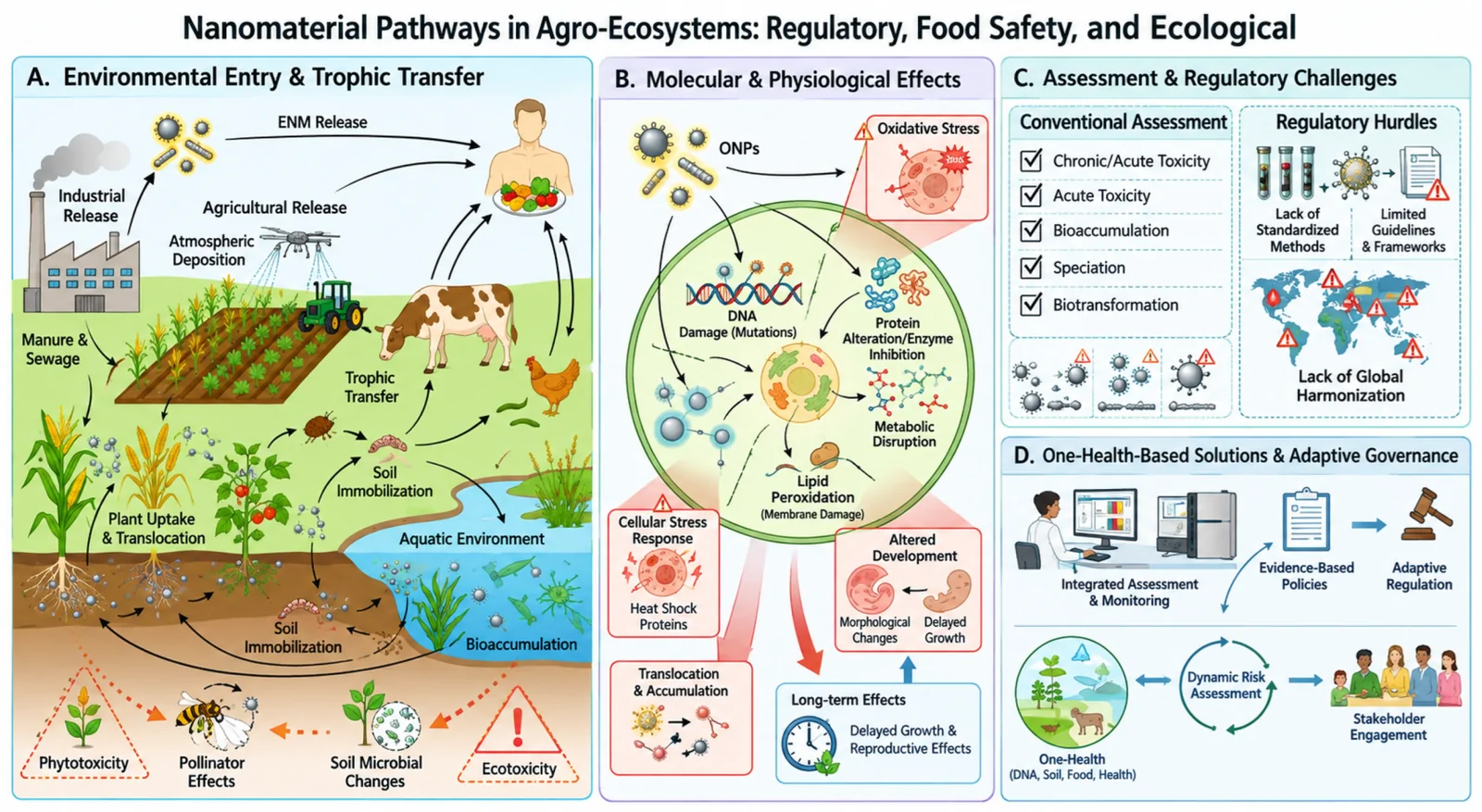
(**A**–**D**) Schematic overview of engineered nanomaterial pathways in agroecosystems, illustrating environmental release, plant uptake, trophic transfer, molecular and physiological effects, regulatory challenges, and omics-driven approaches for ecological risk assessment and governance. The arrows show the path of flow of nanomaterials within environmental compartments, living things, and biological systems. The arrows showing bidirectional or circular flows depict two-way exchanges and feedback loops among molecular responses, ecological processes, risk assessment, and adaptive governance.

Omics technologies provide valuable insights into the molecular responses of plants to nanomaterial exposure and may contribute to future evidence-based risk assessment. However, their application in regulatory decision-making remains challenging because omics responses are highly influenced by plant genotype, developmental stage, environmental conditions, and the inherent physiological plasticity of plants. Consequently, molecular biomarkers should be interpreted together with physiological, agronomic, and environmental observations before being incorporated into regulatory decision-making [185]. The implementation of molecular surveillance alongside ecological surveillance is a stronger strategy for early detection and mitigation capacity. Future regulatory frameworks should integrate molecular evidence with ecological monitoring to improve environmental protection and food safety. Combining the methods of trophic transfer analysis, omics-based risk assessment strategies, and standardized testing protocols will enable a clearer understanding of the basis for transparent, science-based policy development [186,187]. Such integrated approaches support science-based regulation while promoting environmentally sustainable and responsible application of engineered nanomaterials in agriculture (Figure 8).

## 10. Knowledge Gaps and Methodological Bottlenecks

Although considerable progress has been made in explaining interactions between plants and nanomaterials, gaps in knowledge and methodology impede holistic understanding and the translational use of these interactions. A major challenge remains the lack of standardized and environmentally relevant exposure models. Controlled laboratory experiments provide an essential foundation for understanding the mechanisms underlying plant–nanomaterial interactions; however, they cannot fully reproduce the spatial and temporal complexity of agricultural ecosystems. Field responses are influenced by interactions among soil properties, microbial communities, climate, and management practices, making long-term outcomes difficult to predict. Although some engineered nanomaterials have demonstrated beneficial effects under controlled conditions, their long-term performance and environmental consequences under field conditions remain insufficiently understood. Commercial formulations may also contain additional components that influence environmental behavior and biological responses, and these should be considered during future risk assessments. These limitations reduce the ecological relevance of current datasets and restrict reliable environmental risk assessment.

Comparisons among omics studies remain challenging because variability arises not only from analytical methodologies but also from differences in biological and experimental conditions. Plant species, developmental stage, and environmental conditions substantially influence molecular responses to engineered nanomaterials. Additional variation introduced by sampling strategies, sequencing platforms, data normalization, and bioinformatics workflows further complicates data interpretation. The absence of standardized experimental protocols, reference datasets, and curated databases continues to limit reproducibility and cross-study comparisons. Limited integration of transcriptomic, proteomic, epigenomic, and metabolomic datasets continues to restrict the development of comprehensive system-level models of plant responses to nanomaterials. Long-term and multi-generational studies remain limited because of financial, technical, and logistical constraints. Most studies are acute or short-term, providing little information on cumulative, adaptive, and transgenerational impacts. Consequently, delayed phenotypic responses and ecosystem-level feedback remain poorly understood. Similarly, datasets covering multiple developmental stages under realistic field conditions remain limited, restricting predictive modeling.

Reproducibility remains a major challenge because nanoparticle synthesis, characterization, and storage are not yet fully standardized. Variations in the physicochemical characteristics of different engineered nanomaterials, such as Ag NPs, ZnO NPs, TiO_2_ NPs, and CeO_2_ NPs, contribute to differences in biological responses. Incomplete reporting of nanoparticle properties and experimental conditions further limits reproducibility and independent validation. Addressing these challenges will require standardized methodologies, transparent reporting, open data sharing, and coordinated long-term research efforts. Table 6 represents the systems-level outcomes of plant–nanomaterial interactions.

Table 6 illustrates that plant responses to nanoparticles should not be considered as isolated biological events but rather as components of an interconnected physiological network. Changes in nanoparticle uptake and distribution influence cellular signaling, which subsequently affects reactive oxygen species homeostasis, antioxidant defenses, hormone regulation, nutrient acquisition, gene expression, and interactions with the plant-associated microbiome. These responses often occur simultaneously and influence one another, resulting in outcomes that depend on nanoparticle characteristics, exposure conditions, plant species, and environmental factors. Although individual studies frequently focus on a single biological process, integrating these findings reveals that nanoparticle responses emerge from coordinated interactions among multiple physiological pathways. Future investigations that combine molecular, physiological, and ecological approaches will be essential for establishing mechanistic links among these processes and for predicting plant responses under realistic agricultural conditions.

Despite considerable progress in understanding plant responses to environmental nanomaterials, several fundamental aspects of plant biology remain insufficiently resolved. Although many mechanisms have been experimentally demonstrated, their regulatory coordination remains incompletely understood. For example, the factors that determine the selection and stability of epigenetic modifications during nanoparticle exposure remain largely unknown, making it difficult to distinguish transient stress responses from long-term adaptive regulation. Similarly, the mechanisms controlling nanoparticle movement through xylem and phloem tissues, including the factors that define transport efficiency, unloading, and redistribution among developing organs, have not yet been fully established. Important uncertainties also remain regarding the formation of transport pathways across the plant cuticle and the structural features that regulate nanoparticle entry into internal tissues. These knowledge gaps indicate that many current models represent plausible mechanistic frameworks rather than fully resolved biological processes. Addressing these limitations through integrated physiological, molecular, imaging, and long-term field studies will be essential for developing a predictive understanding of plant–nanomaterial interactions under diverse environmental conditions. Future studies should prioritize integrated transcriptomic, proteomic, epigenomic, and metabolomic analyses under standardized field conditions to establish predictive models of plant responses to specific classes of engineered nanomaterials.

## 11. Conclusions and Future Scope

This review summarizes existing knowledge on the environmental exposure of nanomaterials, demonstrating that such exposure has multi-layered consequences for plants, encompassing uptake, molecular reprogramming, metabolic changes, and agronomic outcomes. Integration of transcriptomic, proteomic, epigenomic, and metabolomic evidence across developmental stages provides a system-level understanding of plant responses to engineered nanomaterials. These findings demonstrate that plant responses to engineered nanomaterials extend beyond acute stress signaling and involve coordinated transcriptional regulation, antioxidant regulation, and epigenetic reprogramming, which together determine whether plants undergo adaptive acclimation or growth inhibition. Notably, metabolomic remodeling has become a pivotal junction point between molecular perturbations and functional phenotypes, specifically redox balance and secondary metabolite biosynthesis.

The importance of epigenetic plasticity in repetition and transgenerational effects is also highlighted in the review, and there is a need to assess both heritable and immediate physiological changes. Important gaps remain in understanding long-term field responses and the regulatory mechanisms underlying plant adaptation. Future studies should emphasize standardized experimental protocols, realistic exposure conditions, integrated multi-omics analyses supported by computational modeling, and the identification of biologically relevant exposure thresholds that distinguish adaptive responses from phytotoxic effects across different classes of engineered nanomaterials. High-resolution single-cell omics and multi-generational studies will provide further insight into tissue-specific and heritable regulatory dynamics. Linking molecular responses with plant performance will improve the practical application of nano-enabled agriculture. In general, the integrative, systems-oriented approach advanced in this manuscript provides a strong basis for further developing mechanistic knowledge and responsible innovation in plant nanobiology, ensuring that technological advancement is not only environmentally safe and sustainable but also supports agricultural resilience.

The increasing commercialization of nano-enabled agricultural products reflects the growing confidence in their potential to improve crop productivity, nutrient use efficiency, and sustainable pest management. However, successful commercialization should be accompanied by comprehensive evaluations of long-term environmental safety and ecosystem impacts. Experience with previously transformative technologies has demonstrated that benefits observed during early adoption may not fully capture unintended consequences that become apparent only after widespread and prolonged use. Therefore, the development of nano-enabled agricultural products should be supported by life-cycle assessment, long-term field investigations, environmental monitoring, and transparent regulatory evaluation. Such an approach will help ensure that innovation is accompanied by responsible stewardship, allowing the benefits of nanotechnology to be realized while minimizing potential risks to soil health, biodiversity, and agricultural sustainability.

Current evidence largely provides isolated observations describing changes in microbial taxa following NP exposure, but a comprehensive mechanistic framework linking these microbial shifts to plant physiological outcomes remains limited. Future studies should combine metagenomics, metatranscriptomics, metabolomics, and plant phenotyping to determine whether NP-induced alterations in microbial communities translate into measurable changes in nutrient acquisition, stress resilience, disease resistance, and crop productivity. Such integrated approaches will help move the field beyond descriptive studies toward a predictive understanding of how different classes of nanoparticles influence plant–microbiome interactions across diverse soil types and environmental conditions.

**Figure 9:**
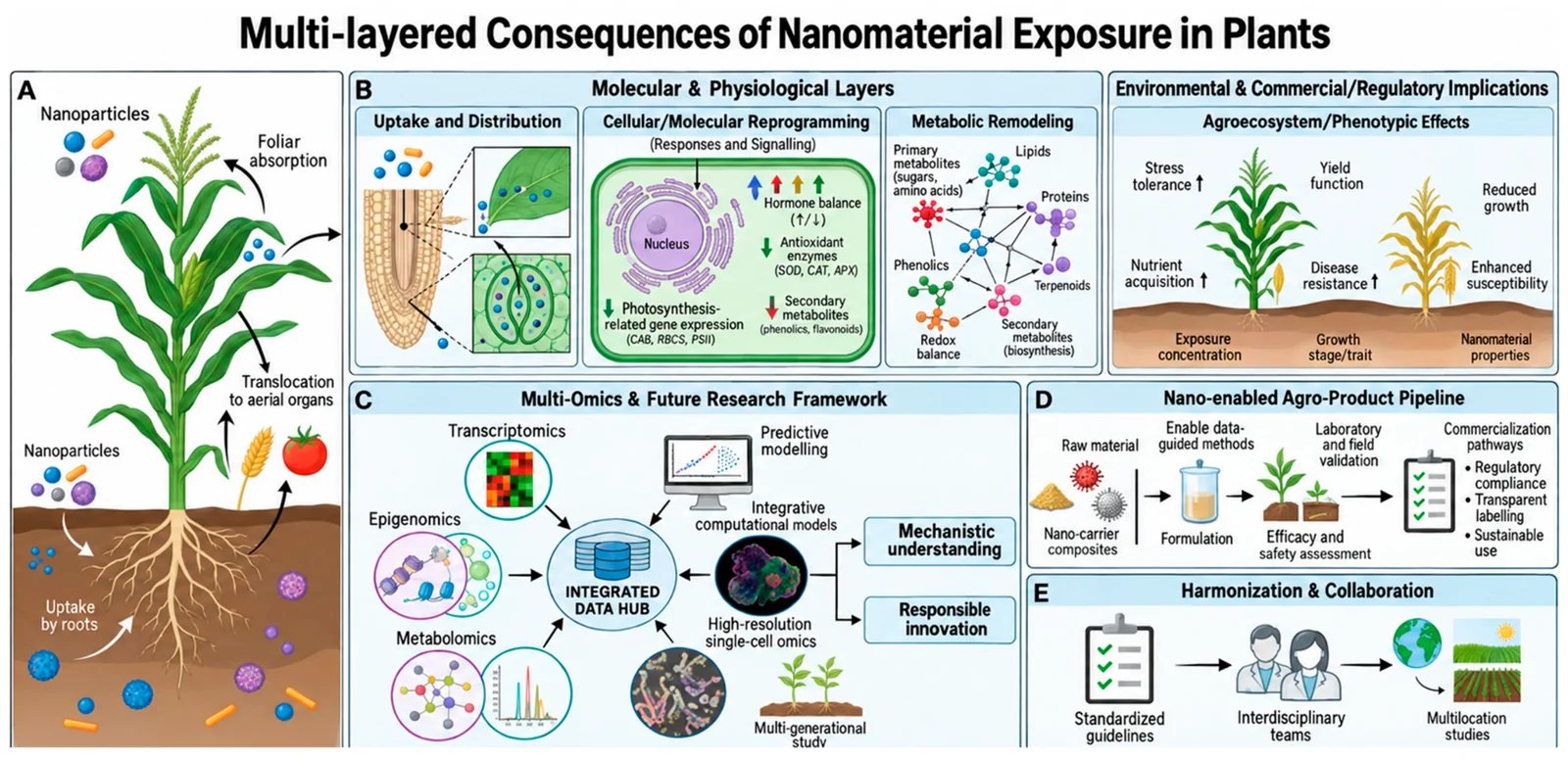
Overview of plant responses to environmental nanomaterial exposure, highlighting uptake and translocation, molecular and metabolic reprogramming, physiological outcomes, and future research priorities for sustainable nano-enabled agriculture. (**A**) Nanoparticles enter plants through roots or leaves and can be transported to aerial organs; (**B**) their exposure induces cellular, molecular, physiological, and metabolic changes. (**C**) Multi-omics integration provides mechanistic and predictive insights into plant responses, (**D**) supporting the development, validation, environmental assessment, and responsible commercialization of nano-enabled agricultural products. (**E**) Harmonization and Collaboration.

Although considerable progress has been made in understanding nanoparticle (NP)–plant interactions, the field remains fragmented, with many studies conducted under different experimental conditions and using diverse nanomaterials, plant species, and soil types. This heterogeneity limits direct comparisons and the development of broadly applicable conclusions. Future progress will require standardized methodologies, coordinated field studies, and interdisciplinary collaboration. Overall, the evidence reviewed indicates that plant responses to engineered nanomaterials cannot be adequately interpreted using isolated physiological or biochemical endpoints. Integrating transcriptomic, proteomic, epigenomic, and metabolomic information across the plant life cycle provides a more comprehensive framework for understanding these interactions. Continued progress in this field will depend on standardized methodologies, environmentally relevant experimental designs, and mechanistic integration across multiple biological scales (Figure 9).

## Figures and Tables

**Table 1:** Early root uptake and cellular entry of ENMs in plants.

Plant Structure	Entry Route/Process	Key Mechanism	Major Molecular Mediator	Driving Force	Governing Nanomaterial Property	Biological Outcome	Reference
Seed coat & imbibition zone	Adsorption to seed coat and penetration through micropyle/microfissures	Regulation of water uptake and ABA–GA signaling	Seed coat, mucilage layer, micropyle	Passive diffusion and imbibition	Particle size, hydrophobicity, surface charge	Germination, dormancy release and radicle emergence	[61,63,75]
Root epidermis/rhizodermis	Rhizospheric adsorption and eco-corona formation	Ligand exchange and eco-corona assembly	Root exudates, root hairs, pectin–cellulose wall	Electrostatic interaction and diffusion	Surface charge, aggregation state, corona composition	Initial bioavailability and root entry	[6,47,75,82]
Root cortex (apoplast)	Cell-wall pore diffusion	Size-dependent apoplastic transport	Cellulose, hemicellulose and pectin matrix	Transpiration-driven flow	Hydrodynamic diameter	Pre-vascular movement	[75,83]
Endodermis/Casparian strip	Apoplast-to-symplast transition	Selective barrier filtration	Casparian strip proteins and suberin lamellae	Barrier-controlled filtration	Particle size, charge	Regulation of vascular access	[75,82]
Plasmodesmata	Cell-to-cell symplastic movement	Dynamic regulation of size exclusion	Plasmodesmata, callose synthase, glucanase, actin–myosin	Symplastic transport	Particle size compatibility and surface chemistry	Intercellular movement	[75,84]
Root apical meristem	Uptake into meristematic tissues	Cell-cycle regulation and cytoskeletal reorganization	Stem cells, PIN transporters, microtubules	Developmental signaling	Surface reactivity and ion dissolution	Altered root architecture	[65,66,67,85]

**Table 2:** Long-distance transport and whole-plant distribution of ENMs.

Plant Structure	Transport Route	Key Mechanism	Major Molecular Mediator	Driving Force	Governing Nanomaterial Property	Biological Outcome	Reference
Xylem	Root-to-shoot translocation	Symplast-to-xylem loading and chelator-assisted stabilization	Xylem parenchyma, tracheary elements, organic acids	Transpirational pull	Colloidal stability and xylem solubility	Distribution to aerial organs	[75,86,87]
Foliar cuticle & stomata	Cuticular and stomatal entry	Surface deposition and diffusion	Cuticle, guard cells, trichomes	Atmospheric deposition and diffusion	Particle size and hydrophobicity	Local and systemic uptake	[8,46,52,88]
Phloem	Source-to-sink redistribution	Companion-cell loading and pressure-flow transport	Companion cells, sieve elements, plasmodesmata	Pressure-flow gradient	Compatibility with phloem sap	Redistribution to developing tissues	[75,89,90,91]
Reproductive organs & seed	Transfer to flowers and seeds	Phloem unloading at reproductive sinks	Embryo sac, pollen tube, unloading tissues	Sink strength	Particle persistence and stability	Effects on seed set and transgenerational transfer	[72,91,92,93]

**Table 3:** Intracellular trafficking and subcellular targeting of ENMs.

Cellular Compartment	Entry/Transport Process	Key Mechanism	Major Molecular Mediator	Driving Force	Governing Nanomaterial Property	Biological Outcome	Reference
Plasma membrane	Endocytic internalization	Clathrin- and lipid raft-mediated uptake	Clathrin, adaptor proteins, small GTPases	ATP-dependent transport	Surface ligands and charge	Entry into endomembrane system	[94,95,96,97]
Subcellular organelles	Endosomal sorting and organelle targeting	Rab GTPase-mediated vesicular trafficking	Rab GTPases, tethering complexes, nuclear pore complex	Membrane sorting	Surface functionalization and particle size	Chloroplast, mitochondrial and nuclear targeting	[98,99,100]
Vacuole	Tonoplast-mediated sequestration	Active transport and compartmentalization	Tonoplast transporters, vacuolar ATPase	Active transport	Dissolution products and chelation potential	Detoxification and intracellular storage	[101,102]

**Table 4:** Multi-omics reprogramming in response to ENMs exposure.

Omics Layer	Specific Regulatory/Molecular Target	Representative Molecular Changes	Key Mediators/Pathways	Analytical/Profiling Approach	Functional/Physiological Implication	Reference
Transcriptomics	Stress-responsive regulons	Upregulation of antioxidant enzyme and redox-modulating genes	WRKY/MYB TFs, heat-shock factors, ROS-responsive cis-elements	RNA-seq, microarray	Activation of detoxification and defense pathways	[19,112]
Transcriptomics	Transporter & metal-homeostasis genes	Differential expression of ABC transporters, ion channels, ZIP/HMA family genes	Metal-responsive transcription factors	RNA-seq, qRT-PCR	Redefined uptake efficiency and intracellular compartmentalization	[7,19,112]
Transcriptomics	Cell-cycle & developmental genes	Reorganization of cyclins, CDKs, meristem-identity genes alongside stress genes	Auxin/cytokinin-responsive TFs	Stage-resolved RNA-seq	Core growth program affected beyond canonical defense response	[66,79,112]
Proteomics	Redox-sensitive protein modification	Reversible cysteine oxidation altering enzyme/TF activity	ROS/RNS, thioredoxin–glutaredoxin systems	Redox proteomics (iodoTMT, OxICAT)	Rapid post-translational adaptation without new protein synthesis	[112,127]
Proteomics	Chaperone/unfolded protein response	Increased HSP and BiP abundance, UPR activation	ER stress sensors, heat-shock factors	Quantitative proteomics, immunoblotting	Maintenance of proteome integrity under destabilizing exposure	[112,122]
Proteomics	Phosphorylation signaling networks	Altered phosphosite occupancy on PIN transporters and scaffold proteins	Receptor-like kinases, MAPK cascades	Phosphoproteomics (LC-MS/MS)	Coordination of uptake, transport, and stress-adaptation signaling	[85,112]
Proteomics	Ubiquitin–proteasome/autophagic turnover	Increased ubiquitination and autophagic flux of damaged proteins	E3 ligases, ATG proteins, 26S proteasome	Ubiquitin-enrichment proteomics, autophagy reporter assays	Proteome renewal; resource shift from growth to maintenance	[112,120]
Metabolomics	Primary carbon/nitrogen metabolism	Redistribution of sucrose/starch flux, altered amino acid pools	Sucrose synthase, invertases, N-assimilation enzymes	GC-MS/LC-MS metabolomics	Growth–maintenance trade-off; altered source–sink allocation	[19,76,112]
Metabolomics	Phenolic/flavonoid biosynthesis	Increased phenolic and flavonoid accumulation	PAL, chalcone synthase, phenylpropanoid enzymes	Targeted/untargeted metabolomics	Enhanced antioxidative defense capacity	[130,131]
Metabolomics	Terpenoid/glucosinolate/alkaloid pathways	Altered precursor flux and biosynthetic enzyme activity	Terpene synthases, glucosinolate biosynthetic genes	Pathway-enrichment metabolomics	Modified plant–herbivore/pollinator/microbe interactions	[130,132]
Epigenomics	DNA methylation dynamics	Locus-specific hyper-/hypomethylation; stable epimutations	DNA methyltransferases, ROS1 demethylase	Whole-genome bisulfite sequencing, methylation-sensitive PCR	Transcriptional accessibility shifts; heritable regulatory variability	[133,134,135]
Epigenomics	Histone modification & chromatin remodeling	Redistribution of activating/repressive histone marks	Histone methyl-/acetyltransferases, redox-sensitive remodelers	ChIP-seq, ATAC-seq	Fine-scale control of detoxification vs. growth gene expression	[99,100,136]
Epigenomics	Small RNA–mediated regulation	Differential miRNA/siRNA accumulation; RdDM recruitment	DICER-like proteins, AGO complexes	Small RNA-seq	Post-transcriptional fine-tuning; TE silencing; mobile stress signaling	[20,93,137]
Phytohormonal networks	Auxin–ABA–ethylene–jasmonate crosstalk	Altered hormone gradients and receptor sensitivity	TIR1/AFB, PYR/PYL, ETR, COI1 modules	LC-MS/MS hormone profiling, reporter assays	Growth–defense balance; root architecture; stomatal and reproductive regulation	[85,125,138]
Systems-level integration	Cross-omics regulatory hubs	Identification of master regulators linking transcriptional and post-translational layers	Network inference, interactome mapping	Multi-omics data integration, machine learning, digital-twin modeling	Predictive modeling of exposure outcomes; rational nanomaterial design	[19,26,139]

**Table 5:** Effects of green-synthesized nanoparticles on seed germination, seedling growth, lant growth, and yield.

Nanoparticle Type	Plant/Crop	Concentration (Optimal)	Effect on Seed Germination	Effect on Seedling Growth	Effect on Plant Growth/Biomass	Effect on Yield/Overall Productivity	Citation
Green-synthesized NPs (various)	Multiple crops	Variable	Enhanced germination & emergence	Improved seedling vigor	Growth promotion	Increased yield in field studies	[21,26,164]
ZnO NPs (Larrea tridentata)	Serrano chili	100–250 ppm	Increased germination % (up to +34%)	Longer roots & shoots, higher biomass	Enhanced seedling development	Not reported	[176]
Fe_2_O_3_ NPs	Basil (various cultivars)	50–200 ppm	Increased GP (up to 95% at optimal doses)	Improved shoot/root length & weight	Positive at low-moderate doses	Not reported	[26,171]
Phytosynthesized Ag NPs	Cucurbitaceae (Bitter gourd, etc.)	75 mM	Significantly enhanced germination rate	Better shoot & root growth	Improved early growth	Potential yield enhancement	[174]
Green-synthesized Ag NPs	Not specified	Optimal priming	+10% higher germination under heat stress	Improved seedling performance	Enhanced heat tolerance	Not reported	[21,174]
ZnO NPs	Various	Low-moderate	Positive induction of germination	Enhanced seedling growth	Improved overall plant growth	Positive impact on yield	[164]
ZnO, Ag, TiO_2_ NPs	Wheat, Rice, Oilseeds	1–100 ppm	Dose-dependent (positive at low conc.)	Increased root/shoot length & vigor	Biomass increases at optimal levels	Yield improvement reported	[164,171]
TiO_2_ NPs	Not specified	High concentrations	Enhanced germination	Positive seedling effects	Growth promotion	Not reported	[79,171]
Green ZnO NPs	Wheat	62 mg/L	Improved germination	+50% root, +105% shoot length	Significant biomass increase	Enhanced productivity	[177]

**Table 6:** Systems-level outcomes of plant–nanomaterial interactions.

Biological Level	Key Molecular/Physiological Modifications	Representative Mediators/Pathways	Analytical/Experimental Evidence Base	Agronomic/Physiological Consequence	Reference(s)
Photosynthesis & light-use efficiency	Altered chlorophyll content, photosystem electron-transport efficiency, and RuBisCO activity, photorespiration, and other processes involved in carbon assimilation and energy metabolism, collectively influencing photosynthetic performance.	PSII/PSI complexes, electron-transport chain, chlorophyll and carotenoids biosynthesis pathway	Foliar nanomaterial application studies linking electron-transport function to pigment content	Changes in carbon-fixation rate and biomass accumulation	[7,88,164]
Stomatal conductance & water relations	Altered stomatal aperture/density, modified transpiration rate	Guard-cell ion channels, ABA signaling, cuticular/stomatal nanoparticle deposition	Stomata-mediated foliar nanoparticle sorption studies	Shifts in water-use efficiency; interaction with drought response	[7,52]
Carbon metabolism & source–sink allocation	Redistribution of fixed carbon toward defense compounds versus growth	Sucrose synthase, invertases, glycolytic enzymes	Resource-allocation theory applied to stress-exposed plants; maturation metabolomics	Growth–maintenance/defense trade-off; altered sink strength	[76,159]
Nitrogen metabolism & protein turnover	Nanomaterial exposure can modify nitrogen metabolism by altering the biosynthesis of amino acids, polyamines, and other nitrogen-containing metabolites, as well as protein turnover and cellular signaling, thereby influencing plant growth, metabolism, and stress adaptation.	Nitrate/ammonium assimilation enzymes, ubiquitin–proteasome system	Targeted proteomic and metabolomic profiling of ENM-exposed crops	Protein turnover shifts; altered availability of signaling molecules	[19,112]
ROS& antioxidant homeostasis	Modulated SOD/CAT/APX activity, MDA/H_2_O_2_ accumulation	Antioxidant enzyme systems, redox-sensitive signaling networks	Comprehensive ROS/RNS/RSS (Reactive Sulfur Species) reviews in plant stress biology; eustress–phytotoxicity studies	Determines cellular damage threshold; eustress versus distress outcome	[127,172]
Phenylpropanoid/flavonoid metabolism	Enhanced phenolic and flavonoid biosynthesis	PAL, chalcone synthase, phenylpropanoid pathway enzymes	Phenolic metabolism–growth relationship studies under stress	Improved antioxidative capacity; modified defense metabolite profile	[130,131]
Terpenoid/alkaloid/hormone-linked specialized metabolism	Altered tocopherol, phytosterol, fatty-acid, and alkaloid profiles	Jasmonate signaling, terpene/alkaloid biosynthetic enzymes	Methyl jasmonate-induced metabolomic shifts in fruit tissue	Altered nutritional/functional quality and ecological interactions	[154]
Phytohormonal signaling networks	Nanomaterial exposure can reshape phytohormone crosstalk involving auxins, abscisic acid, ethylene, jasmonates, salicylic acid, gibberellins, brassinosteroids, cytokinins, and other signaling molecules that regulate plant development and stress adaptation.	TIR1/AFB-, PYR/PYL-, ETR-, and COI1-mediated signaling pathways, together with phytohormone crosstalk, particularly auxin (IAA)–ethylene interactions, coordinate plant developmental and stress responses to nanomaterial exposure.	Phytohormonal-perspective reviews of nanomaterial paradoxical effects	Growth–defense balance; shifts in developmental timing	[125,138,160]
Root system architecture & nutrient acquisition	Changes in lateral root density, root hair proliferation, and meristem activity	PIN auxin transporters, meristem-maintenance gene networks	Root system biology and studies on meristem maintenance and stress responses in meristematic tissues.	Altered soil-exploration capacity; nutrient and water uptake efficiency	[65,66,67]
Reproductive development & yield trait formation	Modulated pollen viability, fertilization efficiency, and phloem unloading to reproductive sinks	Pollen tube growth machinery, phloem unloading transporters	Pollen viability/reproduction reviews; seed phloem-loading studies; stage-specific nanoparticle exposure in rice	Variable seed set, grain/fruit yield, and quality outcomes	[72,79,91]
Soil–plant–microbiome interactions, including the distinct contributions of endophytic and epiphytic microbial communities, which influence nutrient cycling, nanomaterial transformation, and plant responses to abiotic stress.	Rhizosphere microbial community restructuring, altered chemical signaling exchange	Plant growth-promoting bacteria, quorum-sensing molecules, and root-exudate-mediated chemo-signaling	Nanomaterial–plant–microbe interaction studies on growth promotion and stress mitigation	Indirect modulation of nutrient cycling, stress resilience, and productivity	[163,164,166]
Nutritional & functional quality of harvested product	Altered storage protein, lipid, vitamin, and specialized metabolite composition	Maturation-associated metabolic reprogramming	Metabolomic profiling during fruit/seed maturation	Trade-offs among yield, nutritional and functional quality, plant vigor, stress tolerance, and life-history traits, which may vary among cultivars and environmental conditions. quality	[76]

## Data Availability

No new data were created or analyzed in this study.

## Abbreviations

The following abbreviations are used in this manuscript:

ENMs	Environmental Nanomaterials
ROS	Reactive Oxygen Species
ABA	Abscisic acid
ATP	Adenosine triphosphate
NPs	Nanoparticles
